# The Changing Strength and Nature of Fire-Climate Relationships in the Northern Rocky Mountains, U.S.A., 1902-2008

**DOI:** 10.1371/journal.pone.0127563

**Published:** 2015-06-26

**Authors:** Philip E. Higuera, John T. Abatzoglou, Jeremy S. Littell, Penelope Morgan

**Affiliations:** 1 College of Natural Resources, University of Idaho, Moscow, Idaho, United States of America; 2 Department of Geography, University of Idaho, Moscow, Idaho, United States of America; 3 Alaska Climate Science Center, USGS, Anchorage, Alaska, United States of America; Ecole Pratique des Hautes Etudes, FRANCE

## Abstract

Time-varying fire-climate relationships may represent an important component of fire-regime variability, relevant for understanding the controls of fire and projecting fire activity under global-change scenarios. We used time-varying statistical models to evaluate if and how fire-climate relationships varied from 1902-2008, in one of the most flammable forested regions of the western U.S.A. Fire-danger and water-balance metrics yielded the best combination of calibration accuracy and predictive skill in modeling annual area burned. The strength of fire-climate relationships varied markedly at multi-decadal scales, with models explaining < 40% to 88% of the variation in annual area burned. The early 20th century (1902-1942) and the most recent two decades (1985-2008) exhibited strong fire-climate relationships, with weaker relationships for much of the mid 20th century (1943-1984), coincident with diminished burning, less fire-conducive climate, and the initiation of modern fire fighting. Area burned and the strength of fire-climate relationships increased sharply in the mid 1980s, associated with increased temperatures and longer potential fire seasons. Unlike decades with high burning in the early 20th century, models developed using fire-climate relationships from recent decades overpredicted area burned when applied to earlier periods. This amplified response of fire to climate is a signature of altered fire-climate-relationships, and it implicates non-climatic factors in this recent shift. Changes in fuel structure and availability following 40+ yr of unusually low fire activity, and possibly land use, may have resulted in increased fire vulnerability beyond expectations from climatic factors alone. Our results highlight the potential for non-climatic factors to alter fire-climate relationships, and the need to account for such dynamics, through adaptable statistical or processes-based models, for accurately predicting future fire activity.

## Introduction

Fire is increasingly recognized as a key process in the Earth system, impacting large-scale vegetation patterns, carbon cycling, climate change, and human well being [1]. As global climate warms [2], fire activity is projected to increase in many regions [3–6], but the degree to which fire will interact with coincident changes in vegetation and human activities remains unclear. Predicting future fire activity is challenging because the controls of fire regimes vary and interact across multiple scales [7,8]. From interannual to centennial time scales, climate exerts a strong indirect control of fire through its role in shaping patterns of potentially burnable fuels. At seasonal time scales, climate directly influences fire by drying fuels and increasing the probability of fire ignition and spread. Human activity can promote or reduce fire at annual scales, directly through added ignitions or fire suppression, and indirectly at decadal to centennial scales by modifying the composition, abundance, and arrangement of fuels on a landscape [9,10]. Anticipating fire activity under global change scenarios, therefore, depends upon understanding the sensitivity of fire regimes to combined changes in climate, vegetation, and human activity [1,3,4].

**Fig 1:**
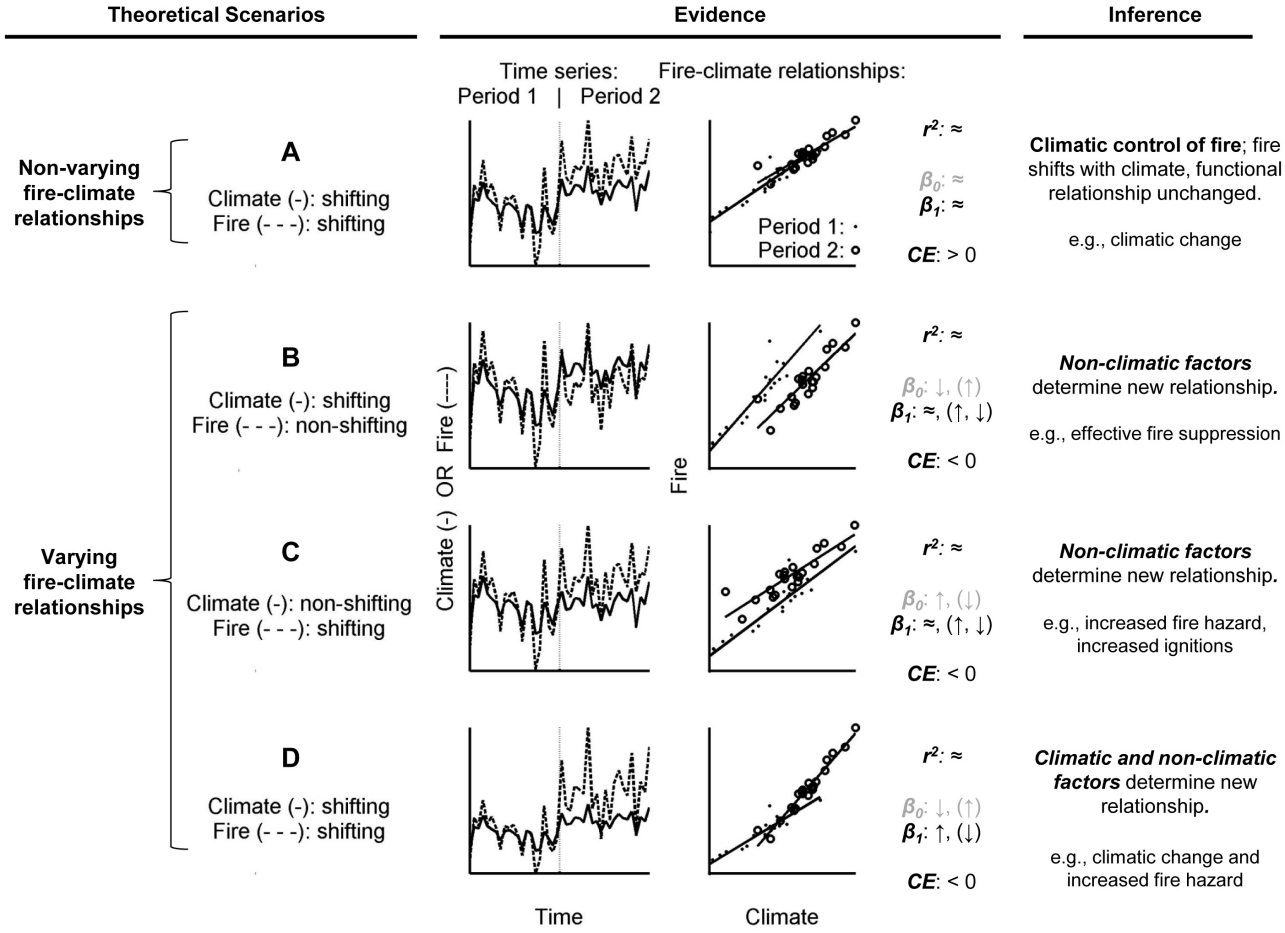
Generalized conceptual model for causes and signatures of shifting fire activity. Scenarios include random variability in “climate” (i.e., a hypothetical metric linked to annual fire activity) which directly determines “fire activity” (e.g., annual area burned or number of large fires). Period 1 is identical in all scenarios, but the y axes are scaled based on values in Period 2. See “Introduction” for a description of each scenario. In all cases of varying fire-climate relationships, a coefficient of efficiency (*CE*) statistic < 0 indicates a lack of predictive skill (for periods outside of the calibration period). β_0_ (intercept) and β_1_ (slope) represent regression parameters; directional changes in parentheses represent hypothetical scenarios not illustrated in the figure.

The interacting controls of fire can be revealed through the time-varying strength and nature of fire-climate relationships. The strength of fire-climate relationships can be measured by the variance in fire activity (e.g., annual area burned, number of large fires) explained by a given climate metric, for example using the *r* ^2^ statistic: high explained variance implies strong fire-climate relationships while low explained variance implies weaker fire-climate relationships, fire activity influenced by factors unaccounted for in a model, or simply little variability to potentially explain. The nature of fire-climate relationships can also change through time, measurable through changing parameters of a linear model predicting log-transformed area burned from a single climate metric. For example, consider a set of hypothetical scenarios where fire activity shifts between two adjacent time periods (Fig 1). If climate was the sole cause of changing fire activity, then the functional link between climate and fire would remain unchanged (Fig 1A). In contrast, if non-climatic or indirect-climatic factors caused the shift, for example through increased human ignitions or fire hazard (natural or otherwise), then the functional relationship between fire and climate would fundamentally change (Fig 1B and 1C). In these scenarios, the new relationship would be less skillful in predicting fire activity in the previous period. The combination of climatic and non-climatic factors could also cause a shift in fire activity, for example through climate change and increased fire hazard, and this scenario would likewise result in an altered fire-climate relationship (Fig 1D).

Observing time-varying fire-climate relationships is difficult, because it requires long-term, high-quality datasets that span periods of fire and climatic variability. Retrospective approaches, for example using tree-ring and lake-sediment records, have revealed changing fire-climate relationships over centennial to millennial time scales, attributable to changing climate variability [11], changing vegetation [12], or changing human land use [13]. If and how fire-climate relationships vary over shorter, decadal time scales is unknown, but such variability could represent an important characteristic of a fire regime itself, and it would have direct implications for extrapolating historical fire-climate relationships to predict future fire activity.

**Fig 2:**
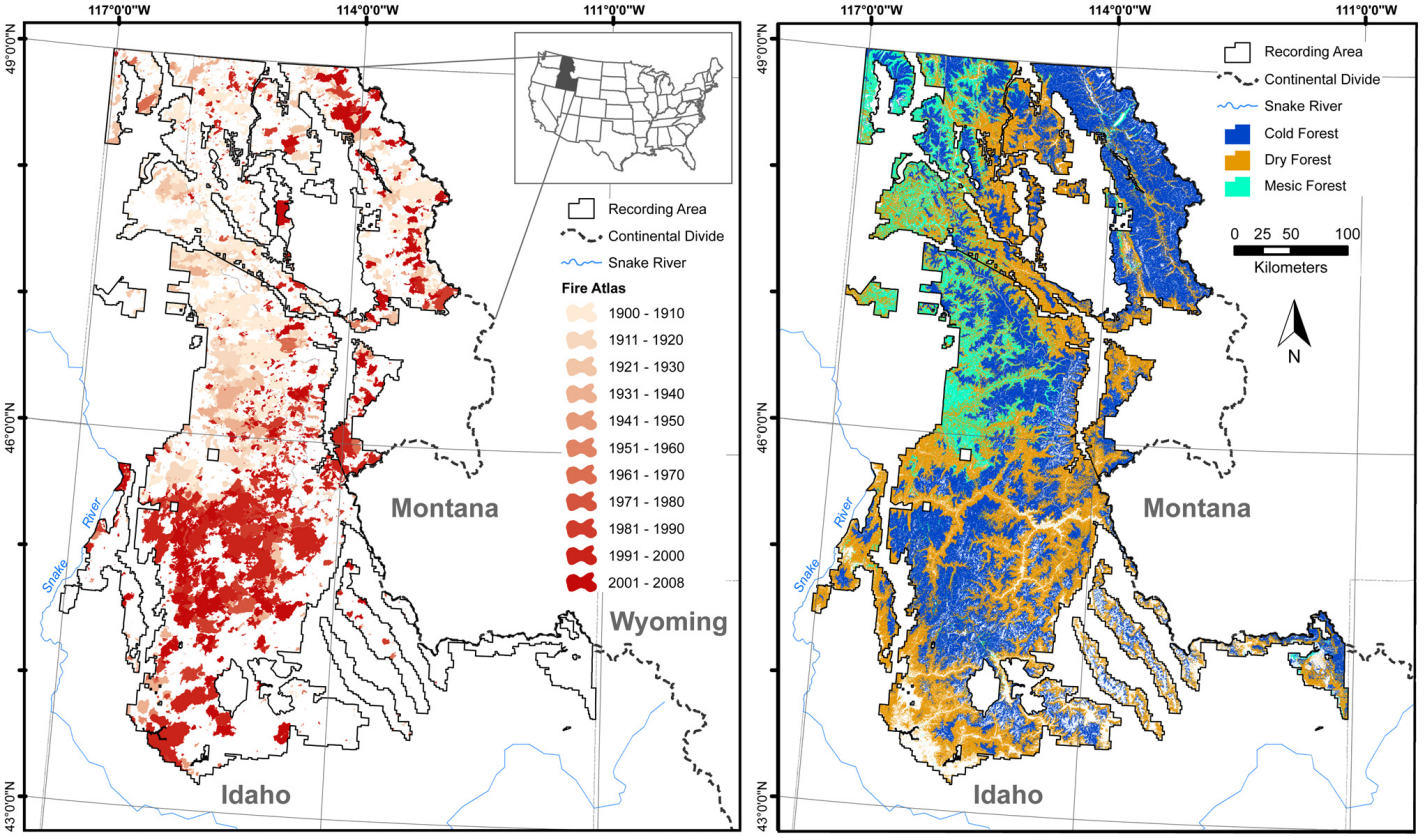
The U.S. Northern Rockies study area in Idaho and portions of Montana, west of the Continental Divide. Area burned, stratified by decade (left), and the three dominant forest types across the study area (right).

In this study, we use the conceptual model outlined above (Fig 1) to evaluate if, how, and why fire-climate relationships varied in one of the most fire-prone regions of the western U.S.A. (“western U.S.”) over the 20^th^ and early 21^st^ centuries (Fig 2). The northern Rocky Mountains of the western U.S. (“Northern Rockies”) are an ideal region to study varying fire-climate relationships because well-documented and widely varying fire activity since the early 20^th^ century coincided with significant variability in climate, land use, and land management [14–18]. Increased forest fire occurrence in the Northern Rockies (since 1985) is well correlated with climatic warming [16,18], and burning in the region accounts for the majority of forest area burned across the western U.S. from 1985–2003 [19]. Consequently, these forests are considered among the most vulnerable to future climate-induced increases in fire activity [6,18,20].

To study regional fire-climate relationships, we develop statistical models using fire-danger, water-balance, and seasonal climate data [21] to predict annual area burned from 1902–2008 in a 120,000-km^2^, mostly forested study area in the Northern Rockies [17]. We identify the key climate drivers of fire activity over the 107-yr period, and then test for changes in the strength and nature and fire-climate relationships over multi-decadal time scales. We interpret our results in the context of existing environmental narratives highlighting land use and land management changes to infer the potential role of climatic and non-climatic drivers of past fire activity. Our results shed new light on the climatic and non-climatic drivers of changing fire regimes and have important implications for predicting future fire activity based on historical fire-climate relationships.

## Materials and Methods

We studied regional fire-climate relationships using a dataset of fire perimeters > 40 ha on federally managed lands in Idaho and areas in Montana west of the Continental Divide [17,22,23], excluding what are non-forested areas south of 43°N latitude (Fig 2). The dataset reflects the compilation of hard-copy and digital polygon fire histories from 12 national forests and one national park, for varying periods spanning 1902–2008. Although unburned areas within fire perimeters are unaccounted for in this dataset [24], we use the term “annual area burned” to approximate the total area burned within fire perimeters in a given year. The final dataset represents a region approximately 40% within the Northern Rockies and 60% within the Middle Rockies ecoprovinces studied by Littell et al. [16] and is dominated by Cold Forest (37%) and Dry Forest (37%) potential vegetation types, with smaller proportions of Shrubland (10%), Mesic Forest (9%), and Woodland (3%; Figs. A and B in S1 Appendix). Our vegetation classification is based on a grouping of Environmental Site Potential (ESP) classes, which broadly identify sites suitable for a given vegetation type, given climate, topography, and substrate [17,25,26]. Using the ESP-based classification is advantageous over a single static vegetation classification, because vegetation is dynamic over multi-decadal time scales, with changes due to, e.g., disturbance, succession, or land use. Although we recognize that conditions have varied over the 107-yr period of analysis, we assume relative differences across the region were nominal: e.g., higher elevations have remained cooler than low elevations, and thus more suitable for Cold vs. Dry forests. While historically fire has likely burned more frequently in Dry vs. Cold forest types [27–30], over the period of our record these dominant forest types burned in proportion to their distribution across the study area [22].

To test if fire-climate relationships differed between the two regionally dominant forest types, we performed our analyses using three domains: the entire study area, Cold Forest, and Dry Forest potential vegetation types. Together, Cold and Dry forests comprise 74% of the study area (across space) and 75% of the total area burned (across space and time). Because spatial autocorrelation of climate anomalies results in well correlated interannual variability in summer temperature and precipitation between Cold and Dry forests (r > 0.95), we use climate metrics spatially averaged across the entire study area in all analyses. If the dominant climate drivers or their strength differ between domains, this would suggest fundamentally different mechanisms controlling burning. Alternatively, the same climate drivers could explain burning in each domain, but the nature of fire-climate relationships could vary, thus indicating differing sensitivity of fire activity to common climate drivers.

**Fig 3:**
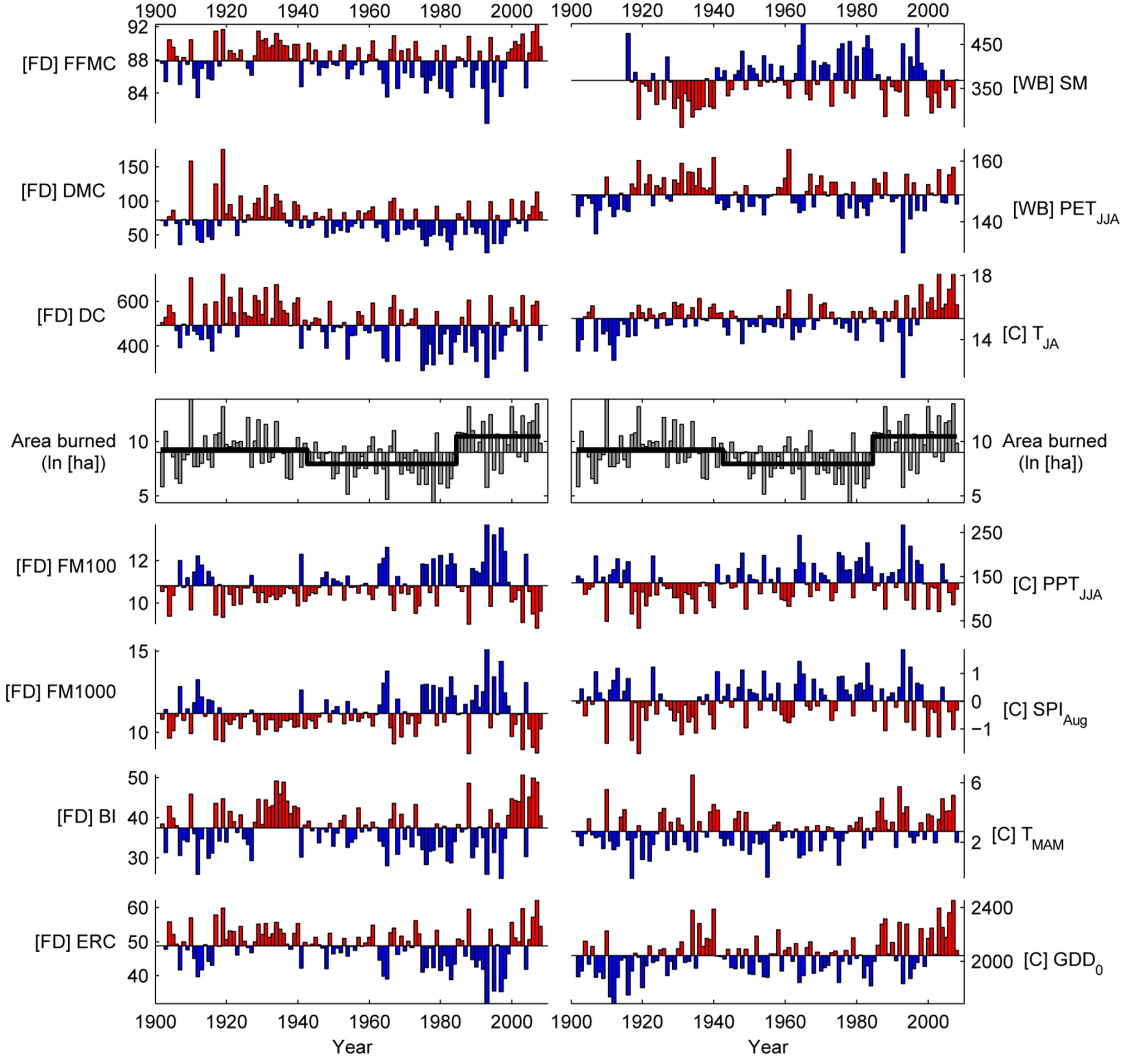
Fire-danger, water-balance, and climate predictors of annual area burned. Series are plotted relative to the series mean, with red (blue) representing above (below) average conditions conducive for fire activity. The center row includes annual area burned (gray), expressed as ln(ha), with average values for each of three periods identified via piecewise linear regression (1902–1942, 1943–1984, 1985–2008). Metrics are organized based on their overall score (upper left to lower right; Table 2). Metric type is identified as “FD” (fire danger), “WB” (water balance), or “C” (climate), with units and temporal definitions listed in Table 1.

We focused on fire-danger and water-balance metrics as potential predictors of annual area burned, but we also used combinations of monthly temperature and precipitation found useful in prior studies [17,18], as well as January-October growing degree days (base 0°C) as a measure of growing (fire) season length (Table 1, Fig 3; S1 Appendix). We considered other climate metrics, including winter temperature and precipitation and the Palmer Drought Severity Index, that would have antecedent influences on fire activity, but we found these less useful for predicting annual area burned. “Fire danger” refers to the static and dynamic elements of the fire environment that influence fire ignition and spread [31], while “water balance” refers to the net tradeoffs between precipitation and evapotranspiration, as influenced by soils and plant water use [32]. Each fire-danger and water-balance metric integrates meteorological variability at different time scales, with characteristic response times of less than one day (e.g., Fine Fuel Moisture Code) to several months (e.g., Drought Code; Table A in S1 Appendix). To derive fire danger and water balance metrics, we used statistically downscaled output from the 20^th^ Century Reanalysis Project [33] and output from the Variable Infiltration Capacity (VIC) model [34]; monthly temperature and precipitation data were acquired from PRISM [35]. All data span 1902–2008 except for soil moisture (output from the VIC model), which were only available from 1916–2008.

**Table 1:** Climate metrics used to predict the natural logarithm of annual area burned.

Category	Predictor metric(s)	Response time	Temporal resolution	Opt. time window
Fire-danger metrics	Fine-fuel moisture code (FFMC)	< 1 day	daily	90-day max.
	Duff moisture code (DMC)	15 days	daily	90-days, 1 Sep.
	Drought code (DC)	52 days	daily	1 day, 1 Sep.
	100-hr fuel moisture (FM100)	5 days	daily	105-day min.
	1000-hr fuel moisture (FM100)	42 days	daily	60-day min.
	Burning index (BI)	30+ days	daily	90-day max.
	Energy release component (ERC)	30+ days	daily	90-days, 15 Sep.
Water-balance metrics	Soil moisture (SM)	> 30 days	daily	30-day min.
	Potential evapotranspiration (PET)	daily	monthly	June-August tot.
Weather / climate metrics	Temperature (T)	NA	monthly	July-August mean
	Precipitation (P)	NA	monthly	June-August tot.
	Standardized precip. index (SPI)	NA	three months	August
	Jan.-October growing-degree days (GDD_0_)	NA	monthly	Jan.–Oct. (forced)

The “optimal time window” defines the within-year period over which each metric is summed or averaged; see “Materials and Methods” for details.

### Statistical analyses and regression models

The goal of statistical analyses was to identify the optimal metrics for predicting annual area burned, both within and outside of 21-yr calibration periods. We also performed analyses with 31-, and 41-yr calibration periods, and our results were generally robust to these variations. We chose the 21-yr period because it represents a compromise between identifying decadal-scale variability in fire-climate relationships and highlighting focal periods from previous studies (e.g., 1987–2003, Westerling et al. 2006), while maintaining a reasonable sample size for regression models. We defined “optimal” using regression statistics, which accounted for accuracy within the calibration period, and a cross-validation statistic, which accounted for predictive skill outside of the calibration period. To satisfy assumptions of normality, the natural logarithm of annual area burned was used in all analyses, commonly done when statistically modeling annual area burned (e.g., [e.g., 16,36,37–39]). Zero values for annual area burned were only present in three instances (< 3%) of the stratified, Cold Forest dataset, and in these cases we added 10 ha to facilitate log transformation; our analyses were not sensitive to this choice (e.g., 10, vs. 1 ha). After log transformation, the annual area burned dataset, and all of the predictor variables, passed a Lilliefors goodness of fit test (α = 0.05) for all (area burned) or 95% (predictor variabiels) of the periods analyzed. We identified the optimal definition for each fire-danger and water-balance metric by comparing statistical summaries (e.g., mean, maximum, minimum) that varied across multiple time scales within a fire season, spanning 1 May through 1 October (e.g., as reported for Northern and Middle Rockies ecoprovinces 1980–2000, [16]; S1 Appendix). This optimal definition was necessary because most fire-danger and water-balance metrics are calculated at daily time scales, yet a single annual value is required for predicting annual area burned. We used these optimal definitions presented in Table 1 in all following analyses.

To rank potential predictors of fire activity, we developed a simple linear model (*y* = *β*_*0*_ *+ β*_*1*_ *x*) or multiple linear regression model (*y* = *β*_*0*_ *+ β*_*1*_ *x +… β*_*n*_ *x*, with *n* = 2 or 3) for each continuous 21-yr calibration period within the dataset (n = 87), where *x* was the climate metric and *y* was the natural logarithm of annual area burned. The first of the 87 periods was centered on 1912 (spanning 1902–1922) and the last was centered on 1998 (spanning 1988–2008). For multiple linear regression models, the maximum variance inflation factor (VIF) of 3.08 was well below a VIF of 10, used to indicate significant multicollinearity in predictor variables [40]. We used the coefficient of determination, *r* ^*2*^ (*R* ^*2*^_*adj*_ for multiple regression models) as a measure of accuracy for each calibration period. We then applied the linear model to predict annual area burned outside of the calibration period (i.e., all years not included in the calibration dataset; n = 86 yr) and used the coefficient of efficiency statistic, *CE*, as a measure of prediction skill [41–43]. The *CE* statistic represents the accuracy of a model (as measured by the mean squared error, MSE, over the calibration period) relative to the null model (measured by the MSE when using the cross-validation period mean as the prediction):

$$CE=1-\frac{MSE(\hat{y})}{MSE({\bar{y}}_{v})}=1-\frac{\frac{1}{N}\sum {\left(y_{t}-{\hat{y}}_{t}\right)}^{2}}{\frac{1}{N}\sum {\left(y_{t}-{\bar{y}}_{v}\right)}^{2}}$$

where *y*_*t*_ is the annual area burned in year *t* of the validation period, *y-hat* is the prediction based on a regression model, and *y*_*v-*_ *bar* is mean annual area burned during the validation period. The *CE* statistic ranges from −∞ to 1, with *CE* > 0 indicating skill: the regression model is a better predictor than the null model. Strong predictors have high accuracy within the calibration period and high prediction skill outside of the calibration period. We therefore used the product of the median *r* ^*2*^ and *CE* to rank potential predictor variables, and we defined “optimal” as the predictor with the highest median product across the 87 periods. Autocorrelation across all predictor and response series was low (i.e., < 0.25 in the majority of series), and our interpretation of changing fire climate relationships does not hinge on associated p-values.

To test for evolving fire-climate relationships through time, we performed all analyses on the entire record (‘global’ analysis), and on the 21-yr time periods from 1902–2008 (n = 87; ‘local’ analyses). We report *r* ^*2*^ (adjusted *R* ^*2*^, for multiple regression models) from the global analyses and plot the time series of regression and cross-validation statistics from each of the 87 local analyses.

### Detecting changing fire-climate relationships and inferring climatic and non-climatic drivers

We used two methods to detect significant changes in fire-climate relationships through time. First, we compared continuous, time-varying calibration accuracy and cross-validation to a null model, where changes in the strength and nature of fire-climate relationships were an artifact of the changing nature of the log-transformed area burned dataset (e.g., mean, variability, range; Fig D in S1 Appendix). The null model presumes the functional link between climate and fire is constant through time, and climate variability, plus a constant error rate representing stochasticity, can explain the variability in annual area burned (S1 Appendix). Given this model, the estimated correlation statistics, model parameters, and cross-validation skill would inherently change through time, simply because of changes in the area burned dataset. We used the central 95% of the observations from 10,000 realizations of this null model as an informal hypothesis test [44]: we interpreted model parameters that remained within this range as consistent with the null model, and parameters that did not as inconsistent with the null model.

Second, we performed a discrete analysis of fire-climate relationships, to help infer the potential mechanisms causing changes across three time periods with varying fire activity, broadly recognized from previous work in the region [16,17]. To identify these three periods objectively, we applied piecewise linear regression to the cumulative log-transformed annual area burned dataset, with the number of break points set to three [45]. To test for significant changes in climate among these three periods, we compared climate data using a Kruskal-Wallis non-parametric analysis of variance and the Chi-square statistic, with an alpha of 0.05. If significant differences were detected, we applied a multiple comparison test using Tukey’s honestly significant difference criterion. Prior to this analysis, each climate metric was evaluated for significant autocorrelation by calculating 95% confidence intervals around lag 1–5 yr autocorrelation values [46]. If there was significant autocorrelation, we used a block-resampling bootstrap technique [47] to estimate the probability of the observed Chi-squared statistic, where the block size was equal to the largest lag with significant autocorrelation. The results of this analysis indicate if and when climate differed among the three time periods. All analyses were conducted using MATLAB software (Mathworks, Natick, MA).

## Results

### Climate predictors of annual area burned

The multiple regression model combining the Duff Moisture Code (DMC) and Growing Degree Days (GDD_0_) yielded the highest accuracy in the study-wide, global analysis, explaining 54% of the variability in the natural logarithm of annual area burned (Table 2). Optimized fire-danger and water-balance metrics alone explained 35–48% of total variability in the natural logarithm of annual area burned, with the DMC (*r* ^*2*^ = 0.460) and Soil Moisture (*r* ^*2*^ = 0.478) as the top univariate models (Table 2). Only once was a metric optimized using a time window that matched distinct calendar months (e.g., June-August for the DMC), and in most cases metrics were optimized using floating time windows among years (Table 1). Time periods summarized maximum or minimum averages over a 30- (Soil Moisture) to 105-day (100-hr Fuel Moisture) period within the fire season, with calendar dates varying among years.

**Table 2:** Results for linear regression models predicting the natural logarithm of annual area burned.

	Total Study Area					Cold Forest					Dry Forest				
	Accuracy		Skill	Score		Accuracy		Skill	Score		Accuracy		Skill	Score	
	*r^2^* or *R^2^_adj_* *		*CE*	*(r^2^_median_* * *CE_median_)*		*r^2^* or *R^2^_adj_* *		*CE*	*(r^2^_median_* * *CE_median_)*		*r^2^* or *R^2^_adj_* *		*CE*	*(r^2^_median_* * *CE_median_)*	
Predictor metric(s)	global	median	median	score	rank	global	median	median	score	rank	global	median	median	score	rank
FFMC	0.351	0.369	0.199	0.046	10	0.350	0.413	0.113	0.037	9	0.329	0.376	0.162	0.044	11
DMC	0.460	0.500	0.294	0.119	2	0.439	0.584	0.175	0.101	2	0.435	0.494	0.265	0.101	2
DC	0.396	0.424	0.219	0.089	4	0.348	0.453	0.095	0.045	7	0.368	0.428	0.172	0.063	6
FM100	0.346	0.371	0.136	0.042	12	0.309	0.388	0.046	0.013	14	0.341	0.429	0.141	0.036	12
FM1000	0.361	0.415	0.114	0.040	14	0.326	0.419	0.000	-0.006	15	0.334	0.407	0.093	0.019	14
BI	0.363	0.374	0.151	0.041	13	0.346	0.397	0.113	0.025	11	0.340	0.363	0.146	0.020	13
ERC	0.380	0.388	0.224	0.066	5	0.352	0.450	0.063	0.025	12	0.371	0.438	0.200	0.061	7
SM	0.478	0.551	0.259	0.102	3	0.448	0.452	0.193	0.081	4	0.448	0.539	0.138	0.077	3
PET_JJA_	0.405	0.418	0.138	0.065	6	0.396	0.498	0.071	0.063	6	0.382	0.401	0.092	0.058	8
T_JA_	0.280	0.263	0.124	0.025	15	0.319	0.410	0.101	0.033	10	0.262	0.248	0.114	0.014	15
P_JJA_	0.355	0.259	0.137	0.042	11	0.339	0.386	0.068	0.024	13	0.360	0.315	0.134	0.049	10
SPI_Aug_	0.373	0.284	0.206	0.048	9	0.356	0.375	0.141	0.041	8	0.387	0.334	0.229	0.057	9
T_JA_, P_JJA_	0.417	0.345	0.224	0.060	7	0.430	0.476	0.199	0.075	5	0.410	0.364	0.220	0.068	5
T_MAM_, T_JA_, P_JJA_	0.482	0.371	0.242	0.055	8	0.488	0.506	0.248	0.093	3	0.483	0.399	0.227	0.074	4
DMC, GDD_0_	0.544	0.483	0.374	0.155	1	0.499	0.590	0.237	0.117	1	0.502	0.476	0.319	0.111	1

Global r^2^ or R^2^adj (for multiple regression models) reflects comparisons between climate metrics and area burned over the entire 107-year dataset. Median *r* ^*2*^ or *R* ^*2*^_*adj*_ reflects the median value from the 86, 21-yr calibration periods, and median *RE* reflects the median value from 86, 86-yr cross-validations prediction periods. The product of median *r* ^*2*^ (or *R* ^*2*^_*adj*_) and median *RE* (“Score”) was used to rank each predictor variable by integrating accuracy (*r* ^*2*^ or *R* ^*2*^_*adj*_) and prediction skill (*RE*).

**Fig 4:**
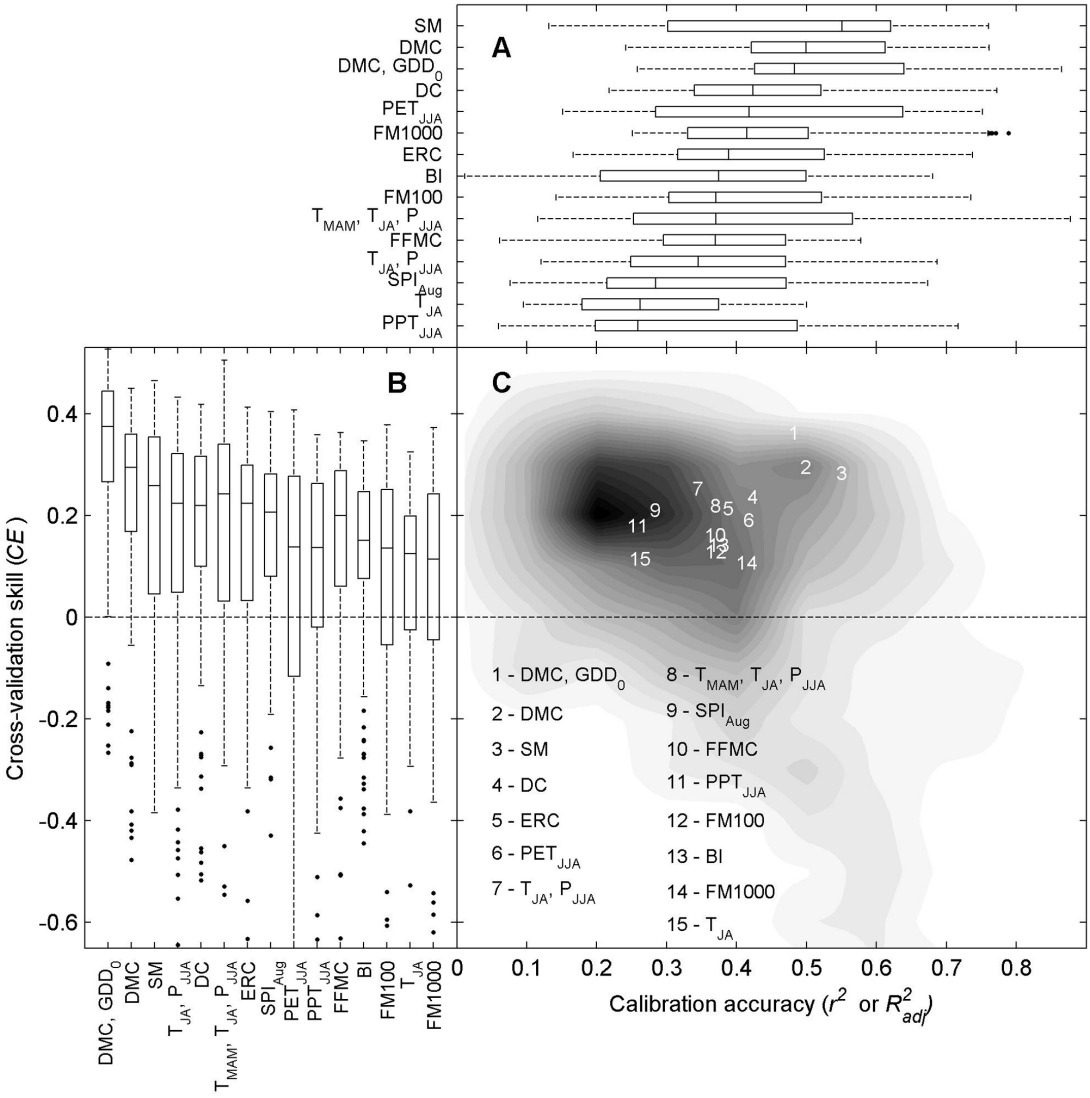
Calibration accuracy and cross-validation skill of potential predictors of annual area burned. (A) Accuracy (*r* ^*2*^ or *R* ^*2*^_*adj*_) from the 87 continuous 21-yr regression models. Metrics are ranked (top to bottom) based on the median value. Boxplots display the median, 25^th^, and 75^th^ quantiles, and whiskers extend to extreme values not considered outliers. (B) Cross-validation skill, *CE*, for all 87 cross-validation periods. Metrics are ranked from left to right based on the median value; *CE* ≤ 0 indicates no predictive skill. (C) Calibration accuracy as a function of cross-validation skill, where darker grey indicates a greater proportion of values. Overall metric rank (i.e., *CE* * *r* ^*2*^ or *R* ^*2*^_*adj*_) is indicated by the within-plot numbers.

Top predictors in the global analysis were also top predictors in the 21-yr, local analyses, lead by the multiple regression model including DMC and GDD_0_, and followed closely by the single-variable models of DMC and Soil Moisture (Table 2, Fig 4C). Calibration accuracy was also high for fire-danger and water-balance metrics, including Soil Moisture, DMC, Drought Code, and June-August Potential Evapotranspiration (PET_JJA_; Table 2, Fig 4A). The multiple regression model including DMC and GDD_0_ had higher accuracy (median *R* ^*2*^_*adj*_ = 0.483) relative to the model combining March-May and July-August temperature with June-August precipitation (T_MAM_, T_JA_, and P_JJA_; median *R* ^*2*^_*adj*_ = 0.371).

All metrics had median *CE* values > 0, indicating predictive skill, but most ranked higher in either calibration accuracy (*r* ^*2*^, e.g., PET_JJA_) or cross-validation skill (e.g., combined T_MAM_, T_JA_, and P_JJA_; Fig 4A and 4B). This tradeoff was also clear in the density distribution of points from all 87 x 15 calibration and cross-validation periods (Fig 4C): models with high calibration accuracy tended to have low cross-validation skill, and vice versa. The exceptions were top three overall models, which ranked 1^st^ to 3^rd^ in both accuracy and skill.

The ranking of the best climate predictors of annual area burned varied little between Cold and Dry forests (Table 2; Figs. A and B in S2 Appendix). The top-performing DMC, GDD_0_ model ranked 1^st^ and the single-variable DMC model ranked 2^nd^ in all analyses. Cross-validation skill was similarly robust, with three of the five top-ranked metrics also in the top five for analyses stratified by forest type. Fire-climate relations were slightly stronger and more sensitive in Cold vs. Dry forests: many top-ranking models had higher median accuracy and/or more extreme regression parameters in Cold Forest (Fig C in S2 Appendix). The variability through time, however was generally similar among the total study area and the two forest types.

### Temporal variability in fire-climate relationships

**Fig 5:**
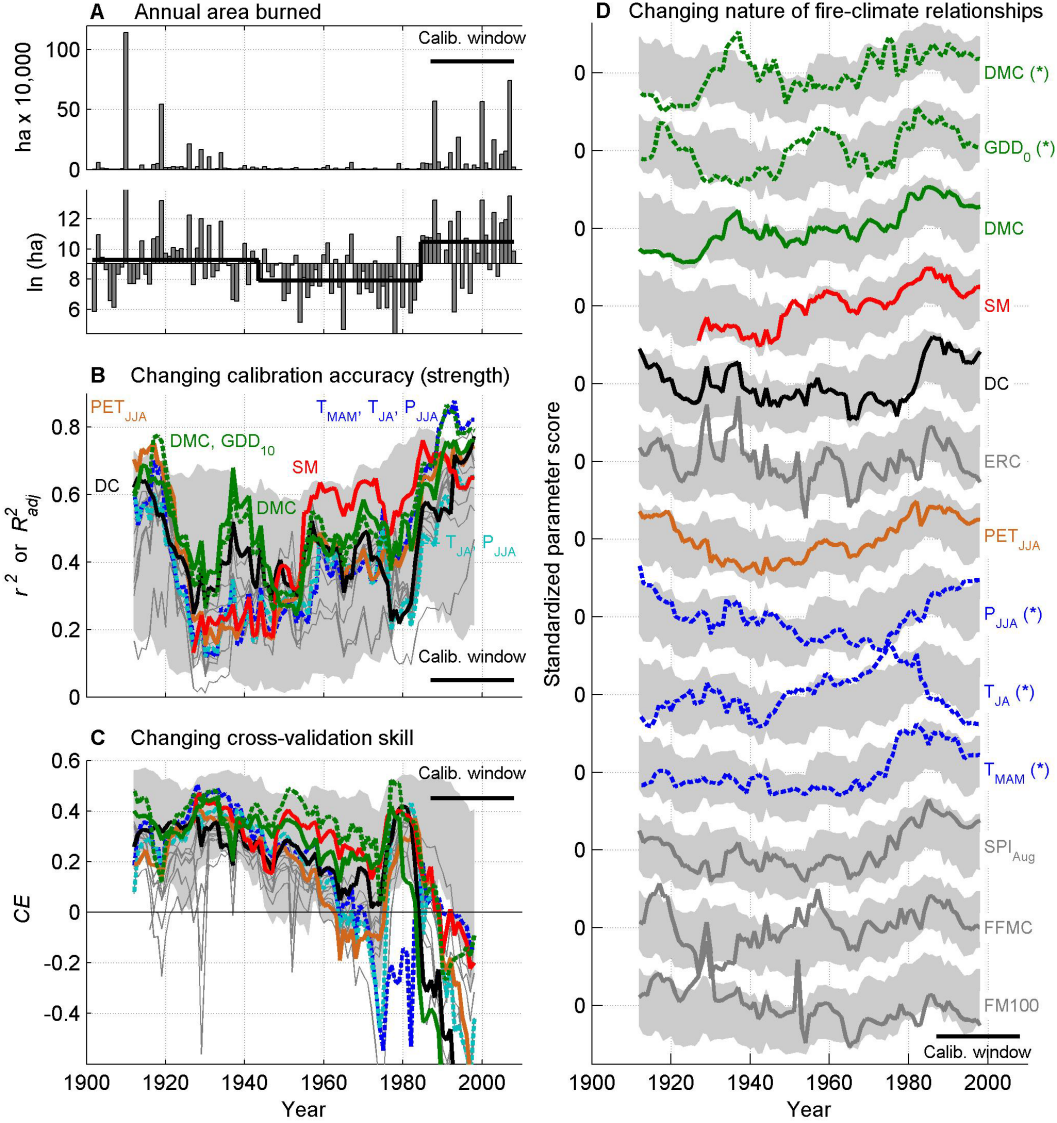
Variability in the strength and nature of fire-climate relationships through time. (A) Annual area burned in linear and log-transformed space (baseline = series-wide average; thick black line = period averages, as in Fig 3). (B) The changing strength of fire-climate relationships (*r* ^*2*^ or *R* ^*2*^_*adj*_) for each 21-yr model. Metrics with the highest explanatory power are labeled, and the length of each overlapping calibration period is represented in the lower right of the panel (“Calib. window”). (C) Changing skill of fire-climate relationships, *CE*, as in (B). (D) Changing nature of fire-climate relationships, illustrated by varying model parameters through time. The y-axis is the standardized β_1_ parameter (for univariate models) or β_1,_ β_2_ or β_3_ parameter for the three-variable models (illustrated by dashed lines), with mean 0, and standard deviation 1. Positive values represent a positive influence on area burned. Metrics are ordered from top to bottom based on the overall model score (Table 2).

Time-varying calibration accuracy and cross-validation skill displayed two overall patterns highlighting variations in the strength and nature of fire-climate relationships (Fig 5): (1) The early 20^th^ century and the decades ca. 1980–2008 were characterized by strong fire-climate relationships, while fire-climate relationships were weaker for much of the mid 20^th^ century; (2) cross-validation skill (*CE*), which was > 0 for most of the record, decreased to < 0 in recent decades. These patterns were robust to analyses performed using only Cold or Dry forest datasets (Fig D in S2 Appendix), and we thus focus on results from the region-wide analysis. Piecewise linear regression identified change points in the log-transformed area burned record at 1943 and 1985, generally robust to analyses stratified by Cold and Dry forests (S2 Appendix). We use these resulting time periods to frame results from our continuous and discrete analyses.

Over approximately the first half of the 20^th^ century (1902 to 1942) area burned was slightly above the 107-yr average, including the record-setting year of 1910, and three other top-10 years in the record (Fig 5A); total area burned was similar in Cold (36%) vs. Dry (33%) forests (Fig A in S1 Appendix). The strongest predictors explained 60–75% of variability in annual area burned, but accuracy decreased for the 21-yr period centered on ca. 1930 (spanning 1920–1940). Top models emphasized seasonal moisture balance (Fig 5B), and exhibited significant cross-validation skill (*CE* > 0; Fig 5C). High correlations and cross-validation skill were consistent with the null model of constant fire-climate relationships, with the possible exception of the noted decreased accuracy across all metrics centered on ca. 1930.

**Fig 6:**
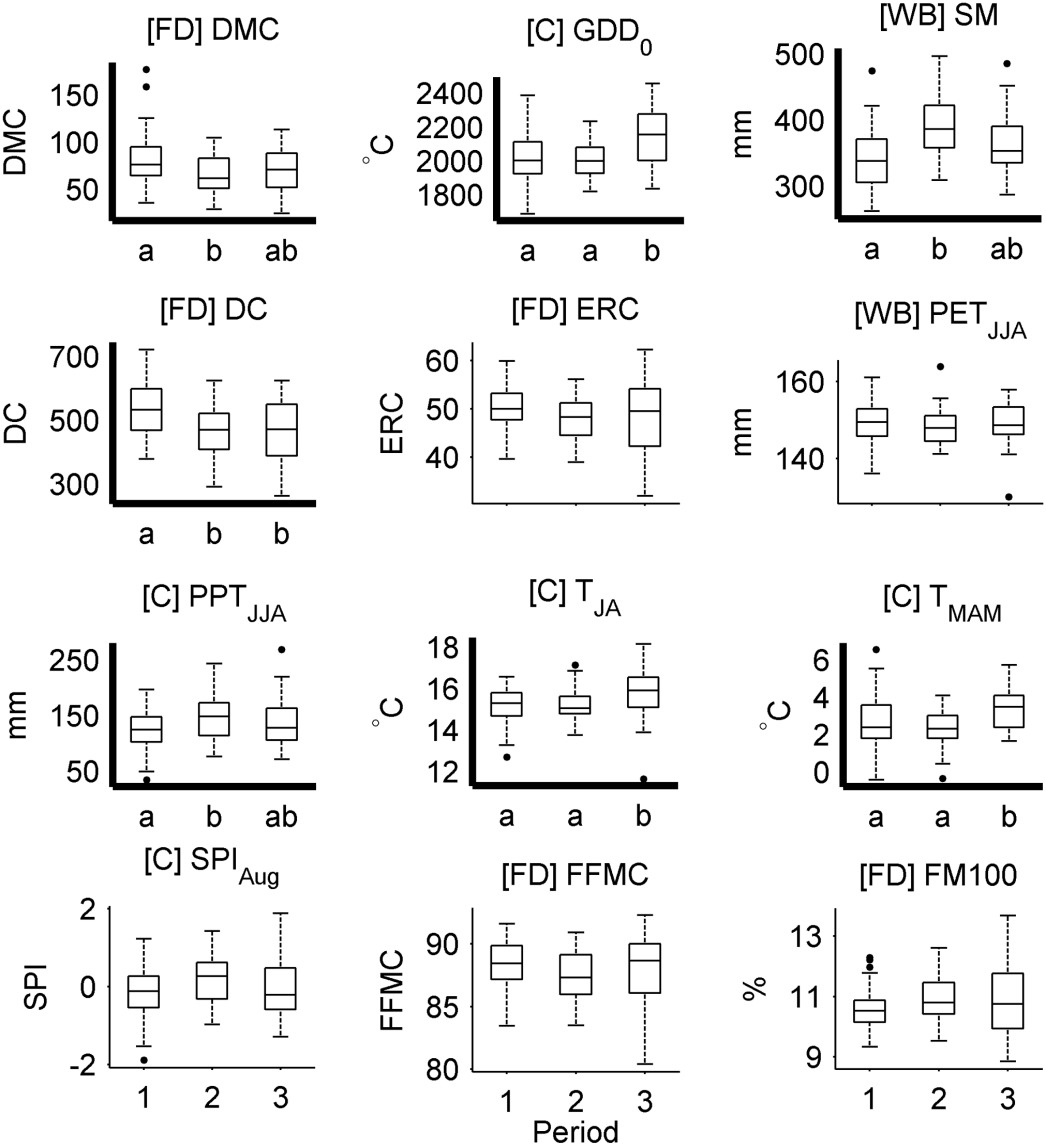
Variability in fire-danger, water-balance, and climate metrics across discrete periods of fire activity. Periods were identified by piecewise linear regression: Period 1 (1902–1942), Period 2 (1943–1984), Period 3 (1985–2008). Metrics are labeled as in Fig 3 and ordered from upper left to lower right based on the overall model score (Table 2). Metrics with significant among-period variability are highlighted with a bold x and y axis, and results of the post-hoc multiple comparison test are illustrated by lower case “a” “b” and “c” below the x axis. See *Detecting changing fire-climate relationships…* for details.

**Fig 7:**
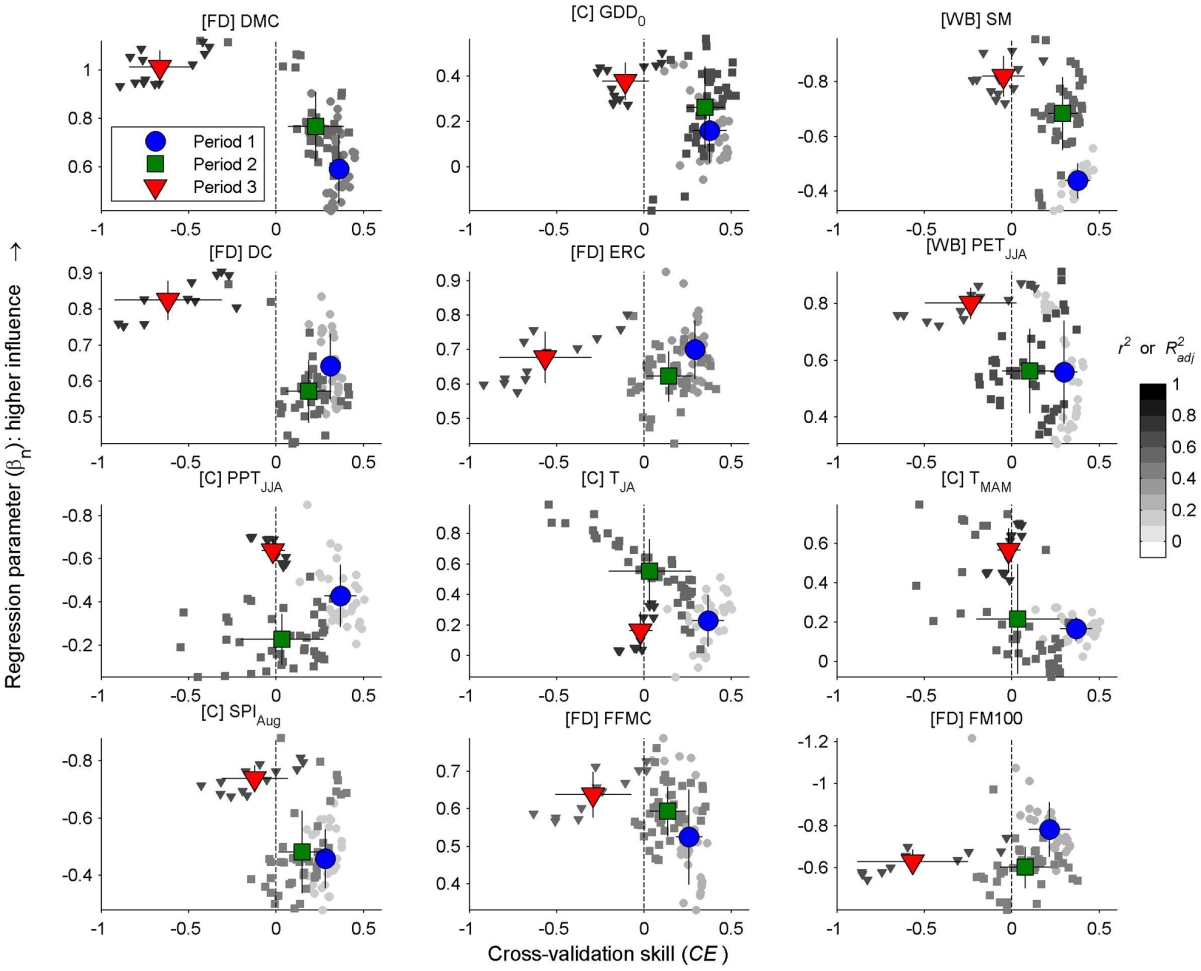
Cross-validation skill, model parameters, and strength of fire-climate relationships for top metrics. Small symbols represent cross-validation skill and the regression parameter for 21-yr calibration windows, stratified by Period 1 (1902–1942, circles), Period 2 (1943–1984, squares) and Period 3 (1984–2008, triangles). The grayscale of each small symbol represents *r* ^*2*^ or *R* ^*2*^_*adj*_ for that window (as in Fig 5B), and large symbols represent the centroid of all values within each period, +/- one standard deviation. Regression parameters represent the slope of the model, β_1_, for single-variable regression models. GDD_0_ represents β_2_ from the combined DMC, GDD_0_ model, while PPT_JJA_, T_JA_, and T_MAM_ represent β_1,_ β_2_, and β_3_ from the three-variable model, respectively. Parameter values indicated the unit (standard deviation) change in log-transformed area burned for a unit (standard deviation) change in the predictor variable: more extreme values indicated a greater influence on annual area burned. Values below the dashed vertical line on the x axis (*CE* = 0) lack cross-validation skill. Metrics are ordered from upper left to bottom right based on the overall model score (Table 2).

The mid-to-late 20^th^ century (1943–1984) was characterized by a shift to below-average area burned, including the seven out of the ten smallest fire years in the record, and not a single year in the upper decile of the record (Fig 5A); more area burned in Dry (42%) vs. Cold (28%) forests (Fig A in S1 Appendix). This shift to decreased burning was associated with significant increases in June-August precipitation, increased Soil Moisture, and decreases in the DMC and Drought Code (Fig 6). Most models explained between ca. 20–50% of the variability in annual area burned, but the DMC and Soil Moisture explained > 60% of the variability for multiple decades (Fig 5B). Regression parameters for top-performing models either changed little (e.g., DMC, Drought Code, GDD_0_, PET_JJA_) or became more extreme (Soil Moisture) relative to earlier the period (Fig 5D and Fig 7). Top-performing models showed predictive skill beyond calibration periods (i.e., *CE* > 0), but *CE* decreased throughout the period, generally in accordance with the null model (Fig 5C). The exception was in PET_JJA_ and the T_MAM_, T_JA_, and P_JJA_ model, which lacked cross-validation skill during the latter part of the period (Fig 5C and Fig 7).

Annual area burned increased distinctly in the mid 1980s and was generally above the series-wide average through 2008 (Fig 5A), and burning was nearly evenly split between Cold (43%) and Dry (40%) forests (Fig A in S1 Appendix). Increased burning was associated with more fire conducive climate: January-October warmth (GDD_0_) increased, at least partially reflecting increased spring (March-May) and summer (July-August) temperatures (Fig 6). Unlike the mid 20^th^ century, the change in climate was not reflected by significant changes in fire-danger or water-balance metrics; although metrics became more fire conducive, they were generally statistically similar to mean values seen in the early 20^th^ century. Thus, the key trend in climate over the past several decades was increased warmth prior to and during the fire season, which would ultimately result in a longer potential fire season. Top-ranking models explained more than 60% and up to 88% of the variability in annual area burned (Fig 5B). Regression parameters for nearly all variables became more extreme (i.e., increased [decreased] for metrics with positive [negative] parameters), in most cases defining new limits relative to earlier periods (e.g., DMC, Soil Moisture, T_MAM_, August Standardized Precipitation Index; Fig 5D and Fig 7). Consequently, cross-validation skill (*CE*) decreased to below 0 for nearly all models during this period. Unlike decreased *CE* values in the late 1970s and early 1980s, *CE* in recent decades exceeded the lower limits expected based on the null model (Fig 5C and Fig 7). Cross-validation skill was low because the unusually extreme model parameters (Fig 7) led to overpredicting fire activity when applied to earlier periods (as in Fig 1D). For example, every model developed using a 1988–2008 calibration period overpredicted annual area burned in earlier periods, by an average of 20% (standard deviation = 4%; Fig F in S2 Appendix).

## Discussion

### Robust climate predictors of annual area burned

Fire-danger and water-balance metrics were robust predictors of annual area burned because they integrate the cumulative, non-linear impacts of temperature, precipitation, vapor pressure deficit, and wind on fuel drying. By doing so on time scales from days to seasons, and even across calendar years (e.g., Soil Moisture), these metrics capture the transition from longer-term patterns in seasonal climate to short-term events, thus accounting for the causal mechanisms linking atmospheric conditions to area burned [8]. Top predictors varied little between forest types and explained as much or more variability (46–54%) in annual area burned across the study period than in similar studies from the western U.S. (e.g., [e.g., 16,36,37,48–50]).

The best predictors of area burned emphasized the importance of annual and fire-season moisture balance for conditioning fuels for fire ignition and spread. Specifically, variables reflected moisture content in the upper and lower layers of the forest litter and soils as a function of variability in precipitation and temperature at monthly and longer time scales (Table 1; [31,51]). Annual area burned in our mostly forested (86%) study area thus appears to be strongly limited by climatic conditions required for fuel drying (i.e., “climate limited”), rather than antecedent conditions required for fuel growth (i.e., “fuel limited”) [16]. This conclusion is consistent with a lack of significant antecedent fire-climate relationships in fire-scarred tree-ring records from Dry forests in the region [27,28] and more contemporary regional analyses spanning the last several decades [21].

### Climate variability and the changing strength of fire-climate relationships

Although climate and fire activity were strongly linked since the early 20^th^ century, fire history in the Northern Rockies clearly illustrates that the strength of fire-climate relationships can vary substantially at multi-decadal time scales. Total explained variance across the study period ranged from < 40% to 88% (Fig 5B), larger than differences documented elsewhere in the western U.S., although in studies analyzing longer, discrete time intervals. For example, Littell et al. [16] found stronger links between climate and annual area burned for the period 1977–2003 relative to 1916–2003 in ecoprovinces across the western U.S. (e.g., *R* ^*2*^_*adj*_ = 0.74 vs. 0.57 for the Northern Rockies ecoprovince), and Miller et al. [36] found stronger links from 1987–2008 (*R* ^*2*^_*adj*_ = 0.54) relative to 1910–1959 (*R* ^*2*^_*adj*_ = 0.34) in northwestern California. Weaker fire-climate relationships in the past could reflect degrading data accuracy, but this is unlikely in our study because model accuracy and skill did not progressively decrease in the past. In contrast, models showed high accuracy and skill during the early 20^th^ century (Fig 5B and 5C).

The varying strength of fire-climate relationships through the mid 20^th^ century can be largely attributed to multidecadal-scale variations in climate, and the subsequent impacts on fuel and soil moisture. The transition from the early to the mid 20^th^ century provides an example of how shifting climate can impact fire activity, when fire is strongly controlled by climate variability (Fig 1A). The overall explanatory power of climate throughout this period is remarkable, given significant changes in land use, land management, and fire suppression [14,15,22,52], and the fact that any errors in the fire and climate datasets would be most prominent during this period (S1 Appendix; [22]). For example, widespread burning during the early 20^th^ century, including record-setting fire years of 1910 and 1919 (ranked 1^st^ and 5^th^), was predictable based on severe moisture deficits (Fig 2 and Fig 5B). The shift to lower fire activity from 1944–1985 was well explained by the shift to less fire conducive climate: the top three predictors all indicated significantly higher fuel and soil moisture (Fig 3), limiting the potential for fire ignition and spread [8]. The mechanisms causing this climate shift are beyond the scope of this study, but this period largely overlaps with the cool phase of the Pacific Decadal Oscillation (1947–1976 [53]).

The clear influence of climate on fire activity does not preclude a role for non-climatic factors in shaping fire regimes, although our analyses provide only limited evidence for such mechanisms. Fire suppression policies, which broadly went into place in the decades after the 1910 fires, and improved fire fighting technologies after World War II [14] may have contributed to lower fire activity during the mid 20^th^ century [17]. However, reduced burning was consistent with and generally predictable from relatively higher fuel and soil moistures during this period, and the observed weaker links between climate and fire were consistent with the null model of constant fire-climate relationships (S1 Appendix; Fig 5B). Fire suppression may explain weak fire-climate relationships observed over shorter time periods, for example ca. 1920–1940 (Fig 5B). Given high moisture deficits throughout most of the 1930s (Fig 3), models predicted higher area burned than indicated by the fire atlas, consistent with successful fire-suppression efforts. However, most effective fire suppression occurred in low-elevation forests, and the pattern of decreased accuracy was observed in both Cold (higher-elevation and more remote) and Dry (lower-elevation and less remote) forests (S2 Appendix). Alternatively, widespread burning in the early 20^th^ century may have reduced fuel loading and/or shifted forest composition to less flammable early-successional species, enough to limit fire ignition and spread [15,54]. Although we lack the data required to test this hypothesis, similar mechanisms have been observed, inferred, or modeled in a range of coniferous forests across North America [54–57].

Finally, we also considered the possibility that weaker fire-climate relationships during the mid 20^th^ century could be an artifact of underreporting in the area burned datasets. However, two patterns suggest that this is unlikely. First, the number of management units reporting fire statistics in the dataset did not systematically decline during this period [22]; and second, reduced burning in the mid 20^th^ century is expressed in several (semi-) independent fire-statistic datasets from across the western United States [16,18,39]. Until a compelling analysis documenting consistent and gross under-reporting of fire during this period emerges, reduced burning in the mid 20^th^ century appears to be a real and robust pattern, consistent with less fire-conducive climate conditions (Figs 3 and 5; [16,24]).

### Unusual fire-climate relationships of the late 20^th^ and early 21^st^ centuries

The Northern Rockies have experienced a pronounced increase in area burned in recent decades (Fig 5A; [17,19]), a trend strongly linked to warming temperatures and ultimately a longer potential fire season, more so than a shift to unusually low fuel and soil moisture (although both were lower than during the mid 20^th^ century; Figs 3 and 6). The importance of early-season warmth is reflected by the increased importance of March-May temperatures and growing degree days in models over recent decades (Fig 5D and Fig 7). This combination of summer moisture deficit and increased fire season length has provided more opportunities for ignition and fire spread and has been a key driver of increased fire activity in the Northern Rockies and across the western U.S. [16,18,20]. Our work further highlights that increased seasonal warmth, particularly in spring, distinguishes the climatic context of recent decades from periods with widespread burning during the early 20^th^ century (Fig 6; although some years were also associated with warm spring temperatures, e.g., 1910, Fig 3; [17]).

Increased fire activity over the late 20^th^ and early 21^st^ centuries is a pattern seen in a number of ecosystems worldwide (e.g., [e.g., 16,18,58,59–61]), although with magnitudes rarely as large and links to warming rarely as strong as in the Northern Rockies. Our results are further distinguished from previous studies because fire-climate relationships have not only strengthened in recent decades, but they have become unique relative to most of the 20^th^ century. Specifically, negative cross-validation skill in recent decades signifies unique fire-climate relationships, relative to earlier periods (Fig 5C and Fig 7), and this pattern implicates non-climatic, indirect-, or non-log-linear climatic mechanisms as contributors to the pronounced increase in area burned in this region. While our work accounts for non-linear relationships, through fire-danger and water-balance metrics and log-linear links to area burned, it does not preclude the possibility that more complicated models (e.g., including threshold effects) could describe a single fire-climate relationship throughout the record. However, the implications of either result would be similar: the nature of fire-climate relationships in recent decades differs from previous decades.

The causes of the unique fire-climate relationships in recent decades are challenging to identify, because we only quantified fire and climatic variables in this study. However, two potential mechanisms can be inferred, based on the fire and climate records themselves, in combination with the conceptual framework outlined in Fig 1 and existing environmental narratives of land use and land management in the region. First, widespread change in the structure and composition of forests, ultimately increasing landscape flammability, is one possible mechanism leading to an amplified response of forest burning to recent climatic change. For example, the relative lack of burning during the ca. 40-yr period from 1943–1984, unusual even in the context of the past 350 years [17,28], would have resulted in a large proportion of landscapes with increasing fire hazard, due to greater fuel loading, higher crown bulk density, and more continuous vertical fuel structures [15,62]. Additionally, logging or land clearing, most concentrated in lower elevations, would have homogenized forests in select areas, potentially creating even-aged stands with increasing fire hazard [62,63]. Combined with the shift to longer, warmer fire seasons since the mid 1980s (i.e., higher fire danger), increased fire hazard would result in greater fuel availability for fire ignition and spread, ultimately leading to greater area burned under a given moisture deficit. This mechanism, invoking interactions between climatic warming and synchronous increases in fuel abundance across landscapes, has been proposed as an explanation for increased burning in boreal forests of Alaska [64,65] and alluded to as a possible consequence of reduced fire activity across much of the western U.S. since ca. 1900 [66]. If true, we would expect to see changing fire-climate relationships in other systems as well, as the legacies of land-use and disturbances interact with ongoing climatic warming.

Second, at finer spatial scales, other non-climatic factors were likely involved in recently changing fire-climate relationships. For example, land use and fire management policies varied widely from the mid to late 20^th^ century, including the creation of wilderness areas (primarily at higher elevations) and the implementation of varying fire use policies [14,15], both reflecting varying human impacts on local vegetation, and fire ignition and spread. The spatial location of burning has also shifted over the 20^th^ and early 21^st^ centuries, with more burning in the Northern Rockies (63%) vs. Middle Rockies (33%) ecoprovince in the early 20^th^ century, with the opposite pattern in the late 20^th^ and early 21^st^ centuries (14% vs. 80%, respectively; Fig B in S1 Appendix). Given the greater sensitivity of Cold vs. Dry forest to a given climate driver (Fig C in S2 Appendix), recent changes in fire-climate relationships could reflect differential burning of Cold vs. Dry forests, but this is unlikely, as nearly equal proportions of Cold and Dry forest burned in the early (36 and 33%) and late (43 and 40%) periods of the record (Fig A in S1 Appendix). Further, neither varying human impacts nor spatially varying forest burning is supported by our analyses: the same pattern of reduced model skill during recent decades is seen in Cold and Dry forests (Fig D in S2 Appendix) and when analyzing records from the southern portion of the study area alone (Fig E in S2 Appendix), where most burning has occurred in recent decades (Fig B in S1 Appendix). Objectively detecting the influence of human activities on observed changes in fire-climate relationships will require conducting analyses at finer spatial scales, and including metrics reflecting human activity.

Finally, changing fire-climate relationships in recent decades could arise from varying accuracy in the climate data, particularly further back in time when observations become limiting. However, as noted earlier, the strong fire-climate relationships in the early 20^th^ century argue against a systematic deterioration of climate (or fire) datasets. To further evaluate the possibility, we tested the structural uncertainty in the downscaled climate data by examining summer precipitation in three high-resolution datasets (Fig C in S1 Appendix). In addition to finding broad interannual agreement across these datasets, the coherent pattern of decreased strength and skill was observed across fire-climate relationships using various metrics (and downscaled climate datasets). Together, these findings suggest that any uncertainty arising from errors in the climate data is relatively small and cannot explain the larger changes in fire-climate relationships we documented. However, in other geographic locations and at smaller scales it may be prudent to consider temporal uncertainty of different fire weather and fire climate estimates as predictors of fire activity.

### Implications for anticipating future fire regimes in forest ecosystems

Despite uncertainties in the cause of recently varying fire-climate relationships, our work suggests that variability in climatic and non-climatic factors can combine to alter links between climate and fire over multi-decadal time scales. If recent trends reflect the combined impacts of climatic warming and increased flammability after 40+ years of unusually low fire activity, then the Northern Rockies may foreshadow how other regions in the western U.S. will respond to the “fire deficit” documented by Marlon et al. [66]. The proposed mechanisms highlight the potential for increased fuel abundance to amplify the response of wildfires to future climatic change, and warrant further study as a potential driver of fire regimes in recent and future decades. The converse is also possible; large increases in annual area burned (e.g., 75–400%) over a multi-decadal period would reduce biomass across broad regions and could weaken fire-climate relationships due to fuel limitation over large scales [20,64,65,67,68]. Together, this body of work increasingly highlights feedbacks among climate, fire, and vegetation as an important mechanism shaping current and future fire regimes.

Changing fire-climate relationships also have practical implications for predicting future fire activity based on historical fire-climate relationships (e.g., [e.g., 3,20,68–70]): by definition, they imply limits in modeling fire activity beyond a given calibration period (with or without climate change). In addition to testing for time-varying fire-climate relationships, we suggest that functional relationships be conditioned not only on vegetation type (e.g., [16]), but also on the (potentially state-dependent) flammability of a given vegetation type, which varies more dynamically as a function of climate, disturbance, and land use. Using functional relationships from diverse climate and fire scenarios may be one way to provide a more realistic range of future scenarios from statistical models. Integrating the mechanisms causing varying fire-climate relationships into process-based models (e.g., [e.g., 5,71,72,73]) will also help account for potentially varying fire-climate relationships in the past and future.

## Supporting Information

S1 Appendix Supplementary Methods. (DOC)

S2 Appendix Supplementary Results. (DOC)
