# Supplementary material for: The Changing Strength and Nature of Fire-Climate Relationships in the Northern Rocky Mountains, U.S.A., 1902-2008
Source: PLoS One. 2015 Jun 26;10(6):e0127563. doi: 10.1371/journal.pone.0127563 (PMC4482589; doi:10.1371/journal.pone.0127563)
Supplement: S1 Appendix — (DOC) [file pone.0127563.s001.doc]

# S1 Appendix: Supplementary Methods

Fire atlas dataset

## The spatial and temporal distribution of burning varied among potential vegetation types (Fig. A), and between the Northern and Middle Rockies ecoprovinces (Fig. B). The time periods used in Figures Aand B were determined based on results, reported in “Results, Temporal variability….”

Downscaled meteorological, fire-danger, and water-balance metrics

Monthly temperature and precipitation data were acquired from PRISM and used to calculate 3- 6- and 9-month Standardized Precipitation Index (SPI) and Palmer Drought Severity Index (PDSI; ultimately not used because of poor performance relative to other metrics). Monthly average snow water equivalent (SWE) and daily total column (top 1.6 m) soil moisture (SM) were acquired from Variable Infiltration Capacity (VIC) model output covering the restricted time period of 1916-2008. PRISM and VIC outputs were spatially averaged over the study area from their native 2.5 arc-sec and 7.5 arc-sec resolutions, respectively.

A complete set of readily available daily surface meteorological variables limited the computation of daily fire danger indices. To circumvent this limitation we statistically downscaled output from the 20^th^ Century Reanalysis (Compo et al., 2011) using the Multivariate Adaptive Constructed Analogues (MACA) method , The 20^th^ century reanalysis provides daily and sub-daily coarse-scale (>200 km horizontal resolution) synoptic meteorological fields including surface temperature (2-m), relative humidity (0.995 sigma hybrid-pressure level), downward shortwave radiation at the surface, 10-m wind velocity and precipitation. MACA uses a linear construct of analog weather patterns using training data from gridded historic surface meteorological data from 1979-2010 to downscale daily coarse scale fields from the 20^th^ Century Reanalysis from 1902-2008. This procedure was used to downscale the primary surface meteorological variables (temperature, humidity, winds, precipitation, insolation) required to calculate fire danger indices and potential evapotranspiration at a 5 arc-sec native resolution for the study area. Unlike PRISM and VIC data that are derived directly from available observations, downscaled datasets contain additional potential errors attributable to both the 20^th^ Century Reanalysis and the downscaling method. To minimize potential error arising from reanalysis, we bias corrected temperature, precipitation and dewpoint temperature downscaled from the 20^th^ century analysis data using PRISM. This allows for the temporal sequences and patterns at the sub-monthly timescale to be retained, but prescribes monthly averages or totals to PRISM data. Additional unresolved error at sub-monthly timescales and originating from winds and insolation may thus contribute, but likely play a lesser role for the variables examined. Downscaled daily surface meteorological data were used to calculate daily reference potential evapotranspiration (PET) using the Penman-Montieth equation for a reference grass surface with zero canopy resistance. These data were also used to calculate the following U.S. National Fire Danger Rating System fire-danger metrics: energy release component (ERC), burn index (BI), 100-hour fuel moisture (FM100), and 1000-hour fuel moisture (FM1000). We used fuel model G (dense conifer stand with heavy litter accumulation) in NFDRS calculations as it represents the main fuel class used in regional fire management and assumed climate class 3 (i.e., 21-day greenup) and slope class of 1 uniformly across the study area. We also applied these outputs using the Canadian Forest Fire Danger Rating System to calculate the fine fuel moisture code (FFMC), duff moisture code (DMC), and drought code (DC) .

To evaluate the potential for time-varying errors in the climate datasets to influence our results, we plotted the relationships between June-August precipitation from 1915-2008, spatially averaged over the Northern Rockies ecoregion (Fig. C). These relationships show strong interannual agreement among three datasets: (i) 4-km PRISM data , (ii) 1/16^th^ degree daily data from Livneh et al. , and (iii) 0.5 degree resolution from the CRU . This suggests relative small structural uncertainties in the downscaled climate data that could potentially cause changes in fire-climate relationships. Although there is a slight drop off in correlations to the CRU data after 1980, PRISM and Livneh et al. remain correlated at r > 0.98, expected, given that much of the same data were used in developing reconstructing such gridded estimates.

### Attributes of fire-danger and water-balance metrics

Attributes of the biophysical fire danger and ecohydrological metrics used in the study are provided in Table A. Federal, state and local fire management agencies in the United States use NFDRS outputs in operational decision-making . The 100- and 1000-hour fuel moisture explicitly correspond to the timescale of exponential decay of dead fuel moisture with respect to the equilibrium moisture content . The ERC is a weather-climate build-up index of potential fire energy intensity, and BI is a proxy for the flame length and difficulty of fire control that additionally incorporates fire spread component by including wind speed. The CFFDRS requires less meteorological data and thus has been more widely applied despite being designed specifically for jack-pine systems that consider canopy throughfall . The CFFDRS tracks surface fuel moisture through the Fine Fuel Moisture Code (FFMC), moisture in the upper organic layer of the forest floor through the Duff Moisture Code (DMC) and moisture in the deeper organic matter through the Drought Code (DC). Previous studies have evaluated the NFDRS and CFFDRS , though a direct comparison is problematic due to fundamental differences in the construction of these systems. Reference evapotranspiration is the environmental demand of an actively growing reference crop with unlimited water availability and, and it considers temperature, humidity, solar radiation and wind speed. Soil moisture in the top 160 cm of the soil column is derived from VIC and is an important factor in the water balance and moisture available to vegetation into the dry season.

### Identifying optimal windows for climate predictors of annual area burned

We performed an optimization that identified the temporal window, defined by the day of year and temporal span, for each climate predictor that best reflected annual area burned. For fire-danger and water-balance metrics we consider semi-monthly intervals (1^st^ and 16^th^ day) from 1 May through 1 October covering 11 different time spans including instantaneous and data averaged over the previous 15- to 150-day periods using 15-day increments . We additionally consider the daily extreme values during the fire season of each year as a predictor variable (e.g., lowest FM100, highest DMC). Similarly, we performed an optimization of monthly climate data that considers time periods from January to October of the fire year and time spans that include the previous 1 to 12 months. From the matrix of temporal windows of each predictor variable we calculate both the correlation coefficient, *r^2^*, during the calibration period, and the coefficient of efficiency, *CE*, as a measure of skill outside the calibration period. This was done using a 21-year moving calibration period across the 107-year record. We define the overall model score as the product of *r^2^* and *CE*, an integrative measure of model accuracy during the calibration period (*r^2^*) and predictive skill outside of the calibration window (*CE*). Finally, we define the optimal calibration window as the time period with the largest median model score (*r^2^*CE*). The resulting time series (Fig. 3, main text) reflect varying temporal windows (Table 1, main text) among metrics, and in many cases varying temporal windows among years for a single metric.

### Null model of constant fire-climate relationships

To evaluate the null model of stationary fire-climate relationships through time, we created 10,000 climate time series which were a constant function of annual area burned, plus random noise. Specifically, we used the following model :

$$\begin{matrix} y=\beta_{0}+\beta_{1}x+\varepsilon\\ \varepsilon=N(0,\sigma) \end{matrix}$$

where *y* was the simulated climate metric, and *β_0_* = 5.0074, *β_1_* = 0.0554, *x* was the natural logarithm of the annual area burned time series, and ε is random number from a normal distribution with mean 0 and standard deviation σ. Given these parameters, we varied σ from 1.8 to 3.0 in increments of 0.2 to obtain overall (global) correlations between climate and the natural logarithm of annual area burned similar to those observed by top models in this study (i.e., 0.35 < *r^2^* < 0.54; Table 1 in main text). The central 95% of values from these 10,000 simulations was used to construct an envelope of potential values under the null model. The general shape of this confidence envelope (Fig. D) reflects the patterns in accuracy, cross-validation skill, and parameter values expected under the null model, and excursions from this envelope suggest excursions from the null model.

# References

1. Daly C, Neilson RP, Phillips DL (1994) A Statistical Topographic Model for Mapping Climatological Precipitation over Mountainous Terrain. Journal of Applied Meteorology 33: 140-158.

2. Abatzoglou JT, Brown TJ (2012) A comparison of statistical downscaling methods suited for wildfire applications. International Journal of Climatology 32: 772-780.

3. Abatzoglou JT (2013) Development of gridded surface meteorological data for ecological applications and modelling. International Journal of Climatology 33: 121-131.

4. Allen R, Pereira LS, Raes D, Smith MD (1988) Crop evapotranspiration - Guidelines for computing crop water requirements. Rome, Italy: Food and Agriculture Organization of the United Nations. 300 p.

5. Deeming JE, Burgan RE, Cohen JD (1977) The National Fire-Danger Rating System –1978. USDA Forest Service, Intermountain Forest and Range Experiment Station. General Technical Report INT-39 General Technical Report INT-39. 63 p.

6. Andrews PL, Loftsgaarden DO, Bradshaw LS (2003) Evaluation of fire danger rating indexes using logistic regression and percentile analysis. International Journal of Wildland Fire 12: 213-226.

7. Brown TJ, Hall BL, Westerling AL (2004) The impact of twenty-first century climate change on wildland fire danger in the western United States: An applications perspective. Climatic Change 62: 365-388.

8. Wotton BM (2009) Interpreting and using outputs from the Canadian Forest Fire Danger Rating System in research applications. Environmental and Ecological Statistics 16: 107-131.

9. Livneh B, Rosenberg EA, Lin C, Nijssen B, Mishra V, Andreadis KM, et al. (2013) A Long-Term Hydrologically Based Dataset of Land Surface Fluxes and States for the Conterminous United States: Update and Extensions*. Journal of Climate 26: 9384-9392.

10. Harris I, Jones PD, Osborn TJ, Lister DH (2014) Updated high-resolution grids of monthly climatic observations – the CRU TS3.10 Dataset. International Journal of Climatology 34: 623-642.

11. Cohen JD, Deeming JE (1985) The national fire-danger rating system: basic equations. USDA Forest Service, Pacific Southwest Forest and Range Experiment Station. GTR-PSW-082 GTR-PSW-082.

12. Van Wagner CE (1987) Development and structure of the Canadian Forest Fire Weather Index System. Forest Service Technical Report 35. Ottawa: Canadian Forest Service.

13. Mölders N (2010) Comparison of Canadian Forest Fire Danger Rating System and National Fire Danger Rating System fire indices derived from Weather Research and Forecasting (WRF) model data for the June 2005 Interior Alaska wildfires. Atmospheric Research 95: 290-306.

14. Elsner MM, Cuo L, Voisin N, Deems JS, Hamlet AF, Vano JA, et al. (2010) Implications of 21st century climate change for the hydrology of Washington State. Climatic Change 102: 225-260.

15. Littell JS, Oneil EE, McKenzie D, Hicke JA, Lutz JA, Norheim RA, et al. (2010) Forest ecosystems, disturbance, and climatic change in Washington State, USA. Climatic Change 102: 129-158.

16. Abatzoglou JT, Kolden CA (2013) Relationships between climate and macroscale area burned in the western United States. International Journal of Wildland Fire 22: 1003-1020.

17. Neter J, Kuutner MH, Nachtsheim CJ, Wasserman W (1996) Applied Linear Regression Models. Boston: McGraw-Hill. 720 p.

**Table A:** **Attributes of fire-danger and water-balance metrics used in the analysis.** Fire-danger metrics are from the Canadian Forest Fire Danger Rating System (CFFDRS) and U.S. National Fire Danger Rating System (NFDRS). Water-balance metrics include Soil Moisture, from the Variable Infiltration Capacity (VIC) model. Abbreviations: *MAX (maximum daily value of *), *MIN (minimum daily value of *), PPT (daily total precipitation), RH (relative humidity), DWRS (downwelling shortwave radiation at the surface), T (temperature), V (vapor pressure deficit).

| **Variable** | **Meteorology Inputs** | **Time scale/lag** | **Sensitivity** | **Biophysical Utility** |
| --- | --- | --- | --- | --- |
| **CFFDRS, under closed forest canopy (Van Wagner, 1987)** | | | | |
| FFMC | TMAX, RHMIN, V, PPT | < 1 day | V, PPT, RH | Moisture content of surface fuel, ease of fire ignition. |
| DMC | TMAX, RHMIN, PPT | 15 days | PPT, RH | Moisture in the upper organic layer. |
| DC | TMAX, PPT | 52 days | PPT, RH | Moisture content of deeper layer of organic matter, fire potential. |
| **NFDRS Fuel model G, (Deeming 1977)** | | | | |
| FM100 | All, except V | 5 days | RH, PPT | Moisture content of 100-hour* time lag fuels. Dead fuels in the 2.5- to 7.6-cm diameter class. |
| FM1000 | All, except V | 42-days | RH, PPT | Moisture content of 1000-hour* time lag fuels. Dead fuels in the 7.6- to 20.3-cm diameter class. |
| ERC | All, except V | 30+ days | RH, PPT | Available energy per unit area at flaming front of fire, build-up index of potential daily fire intensity and composite fuel dryness. |
| BI | All | Weather and 30+-day components | V, RH, PPT | Proxy for the flame length and difficulty of fire control. |
| **Water-balance** | | | | |
| PET Penman- Montieth | TMAX, TMIN, RHMAX, RHMIN, DWRS, V | Daily | V, DWRS. | Atmospheric moisture demand from well-watered and growing reference grass surface. |
| Soil Moisture | All (VIC) | > 30 days | PPT, PET, T | Transpirable water for vegetation. |

*denotes exponential decay time of fuel moisture with respect to the equilibrium moisture content.


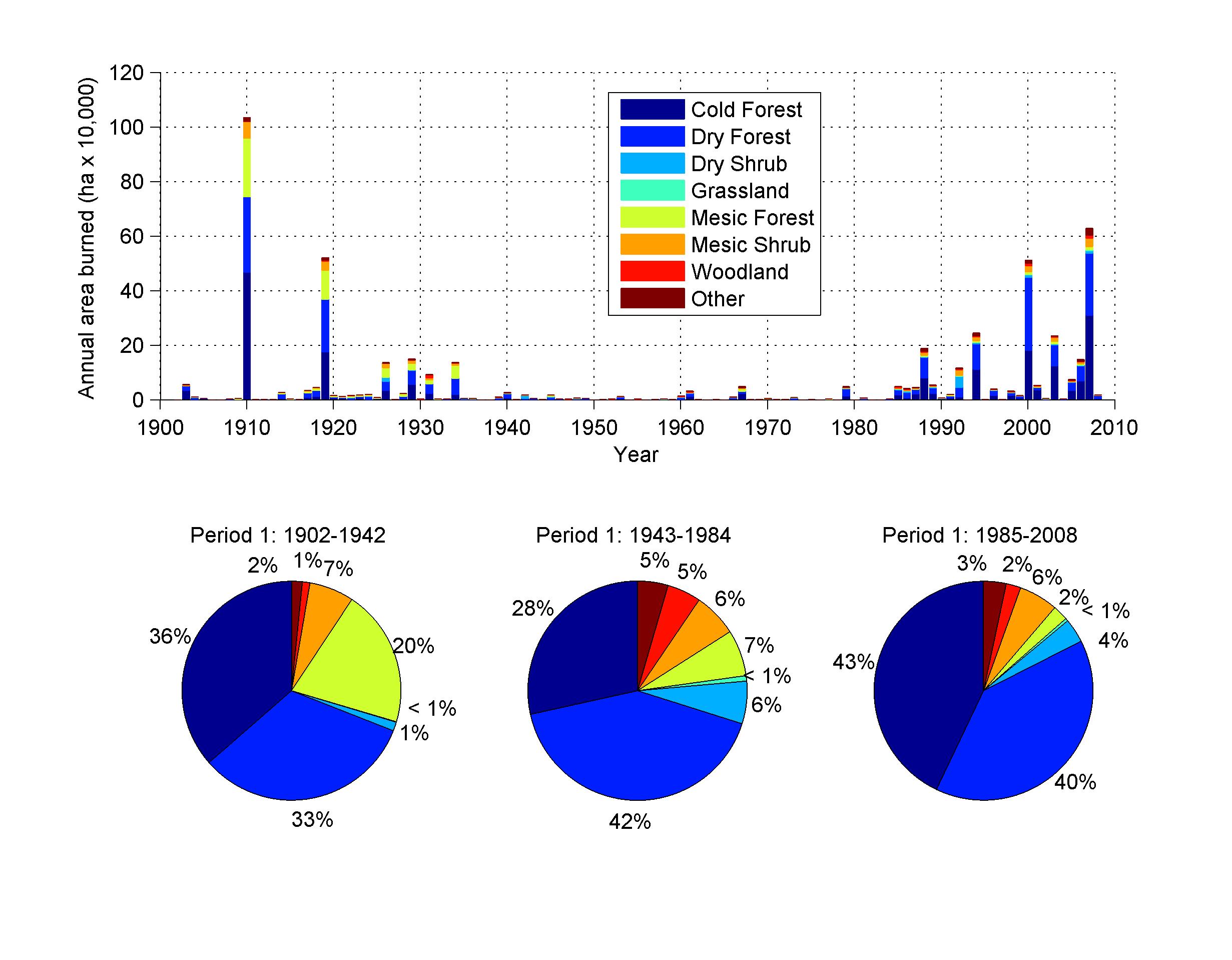


**Figure A. Annual area burned, stratified by potential vegetation type.** Time periods used in the bottom row are defined in the main text (“Results, Temporal variability…”), and the spatial distribution of Cold and Dry forests is illustrated in the main text, Figure 2.


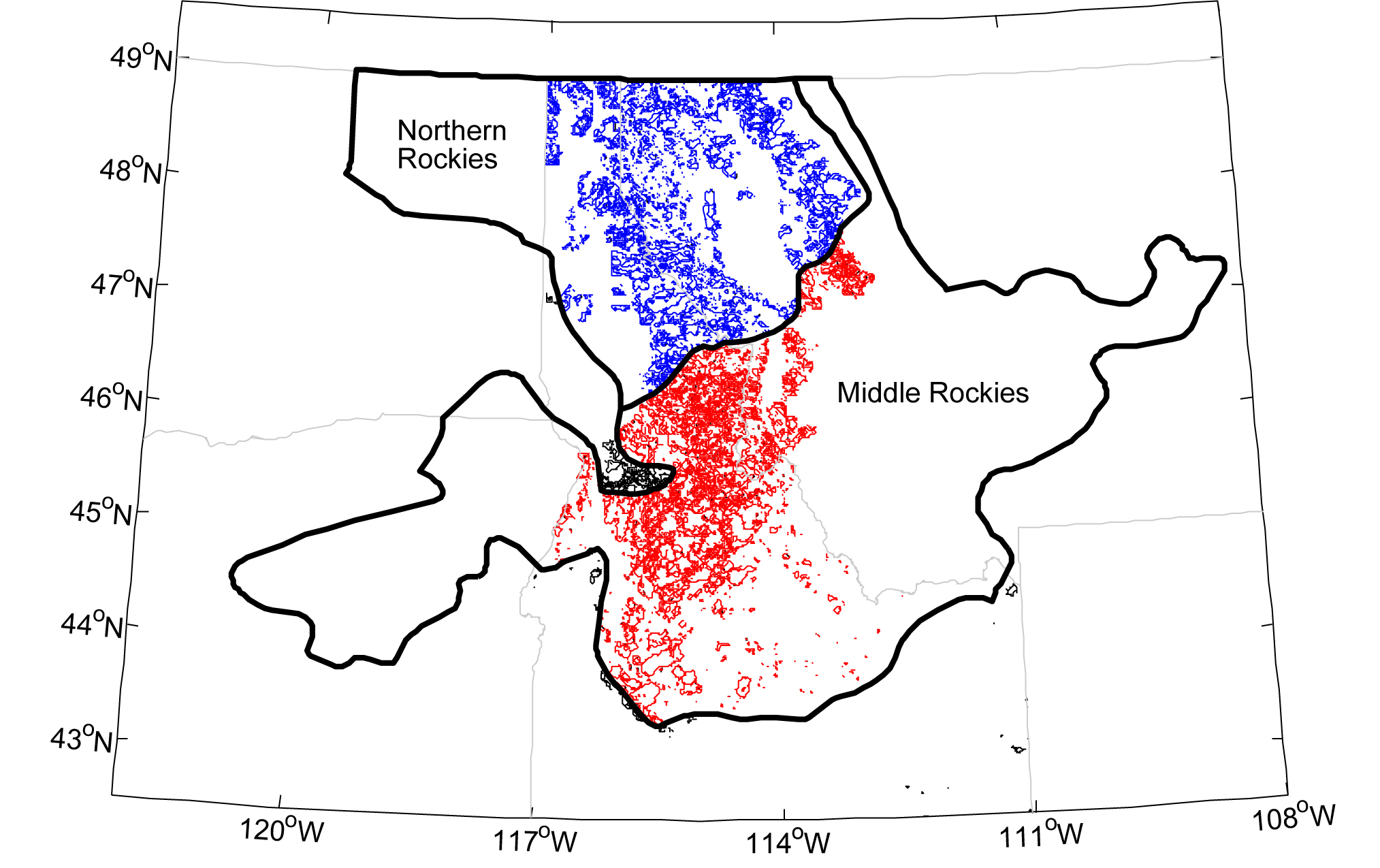
**
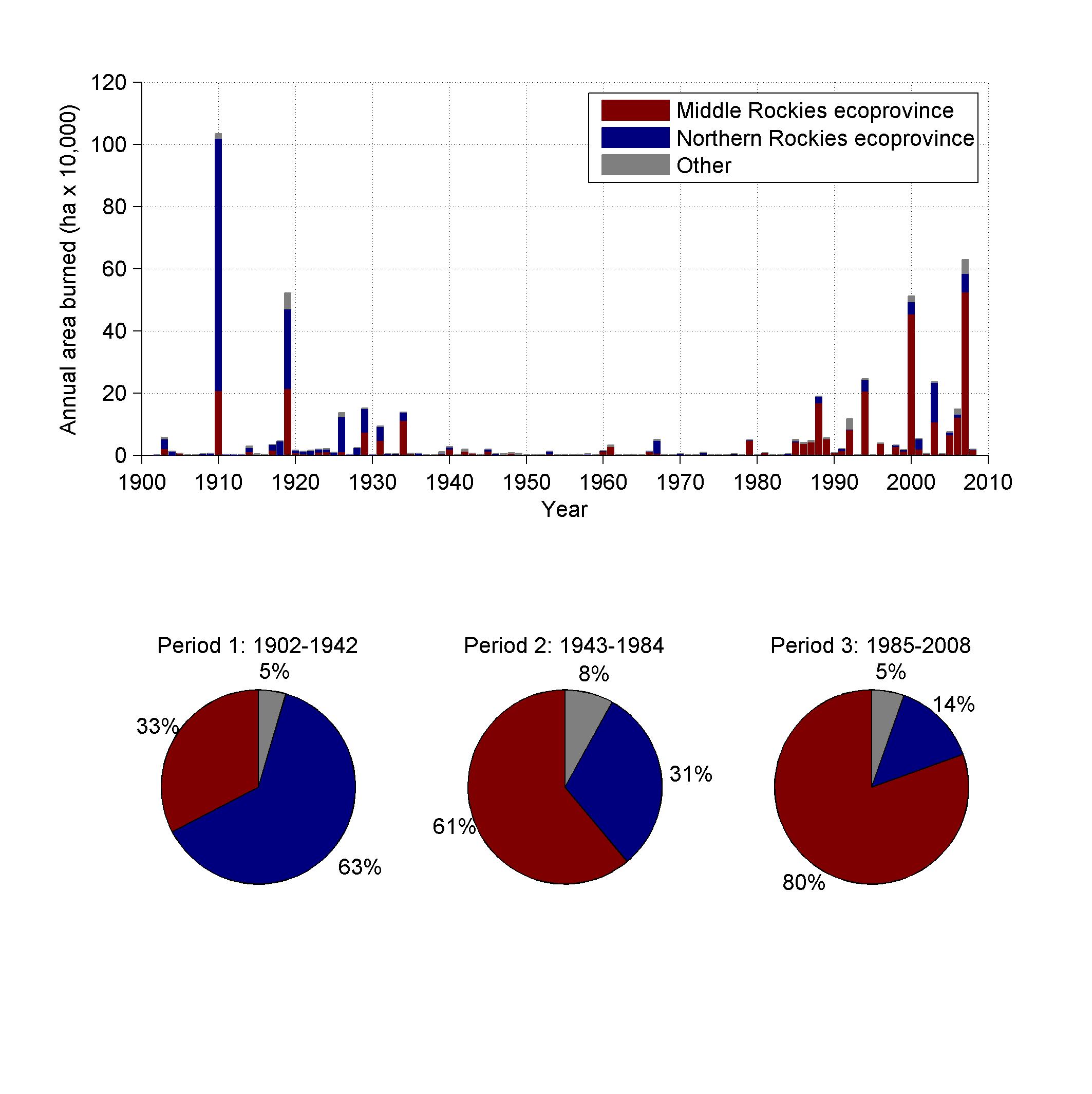
**

**Figure B. Annual area burned, stratified by ecoprovince.** Time periods used in the bottom row are defined in the main text (“Results, Temporal variability…”).

**Figure C. Relationship between summer precipitation from three downscaled climate datasets.** Top panel: Time series of June-August precipitation from CRU , LIVNEH , and PRISM datasets, expressed as percentages of the 1971-2000 normal. Bottom panel: Correlation coefficient between the same climate time series in the upper panel, when compared in overlapping, 30-yr time periods.

**
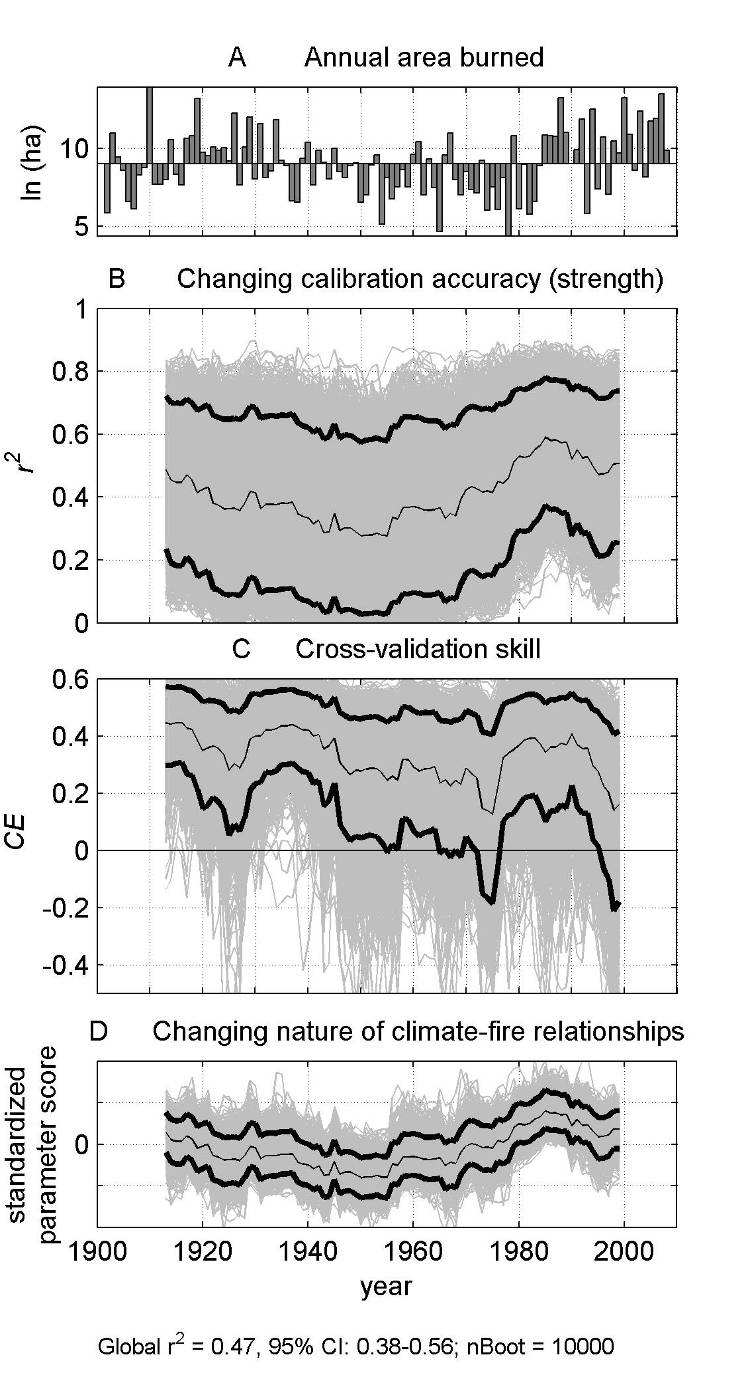
**

**Figure D. Results from 10,000 versions of the null model of stationary fire-climate relationships.** (A) Annual area burned on a log-transformed scale (with a baseline equal to the series-wide average). (B) The changing strength of fire-climate relationships, illustrated by varying coefficients of determination (*r^2^*) for each 21-yr model predicting log-transformed annual area burned. (C) The changing skill of fire-climate relationships, illustrated by varying values coefficients of efficiency (*CE*). (D) The changing nature of fire-climate relationships, illustrated by varying model parameters through time. The y-axis is the standardized slope parameter (mean 0, standard deviation 1). The thick black lines in panels B-D bound 95% of the simulations and are plotted as grey envelopes in the in Figure 5 in the main text.
