# Supplementary material for: The Changing Strength and Nature of Fire-Climate Relationships in the Northern Rocky Mountains, U.S.A., 1902-2008
Source: PLoS One. 2015 Jun 26;10(6):e0127563. doi: 10.1371/journal.pone.0127563 (PMC4482589; doi:10.1371/journal.pone.0127563)
Supplement: S2 Appendix — (DOC) [file pone.0127563.s002.doc]

# S2 Appendix: Supplementary Results

## Climate-fire relationships in Cold Forest, Dry Forest, and Middle Rockies ecoprovince

Piecewise linear regression identified change points in the log-transformed area burned record that were literally or nearly identical to those identified using the entire record: 1943 and 1985 for Dry Forest, 1943 and 1983 for Cold Forest, and 1943 and 1985 for the Middle Rockies ecoprovince. For comparison to Fig. 4 in the main text, we plotted calibration accuracy and cross-validation skill of potential predictors of annual area burned in Cold Forest (Fig. A) and Dry Forest (Fig. B) vegetation classifications individually. The ranking of metrics in these figures is also shown in Table 2 in the main text. To illustrate variability in nature of fire-climate relationships in Cold and Dry forests, we plot the regression parameters through time, for the total study area and stratified by dominant forest type (Fig. C). Finally, for comparison to Fig. 5A-5C in the main text, we plotted variability in the strength and cross-validation skill of fire-climate relationships through time, stratified by dominant forest type (Fig. D), and for the Middle Rockies ecoprovince (Fig. E), where the majority (80%) of area burned has occurred in recent decades.

## Cross-validation examples

To evaluate the nature of low cross-validation skill in recent decades, we plotted predicted annual area burned as a function of observed annual area burned, for the calibration and cross-validation period (Fig. F). These analyses were done for each consecutive year in the dataset, but Figure F represents only a subset of these analyses, staggered by 10 years.


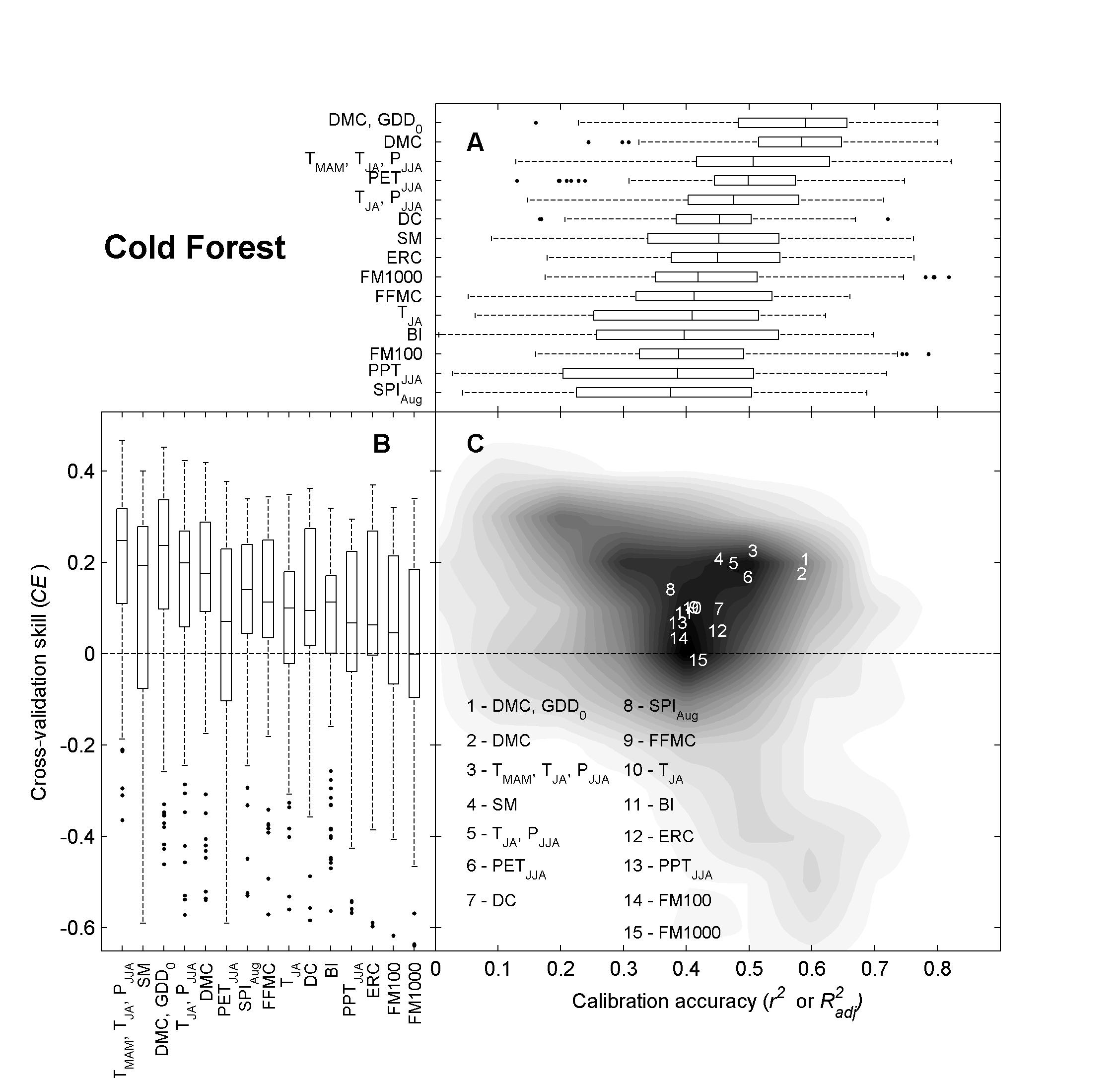


**Figure A. Calibration accuracy and cross-validation skill of potential predictors of annual area burned in Cold Forest sites.** (A) Accuracy (*r^2^* or *R^2^_adj_* for multiple regression models) from the 107 continuous 21-yr regression models. Metrics are ranked (top to bottom) based on the median value. Boxplots display the median, 25^th^, and 75^th^ quantiles, and whiskers extend to extreme values not considered outliers. (B) Cross-validation skill, *CE*, for all 107 cross-validation periods. Metrics are ranked from left to right based on the median value; *CE* < 0 indicates no predictive skill. (C) Calibration accuracy as a function of cross-validation skill, where darker grey indicates a greater proportion of values. Overall metric rank (i.e., *CE* * *r^2^* or *R^2^_adj_*) is indicated by within-plot numbers.


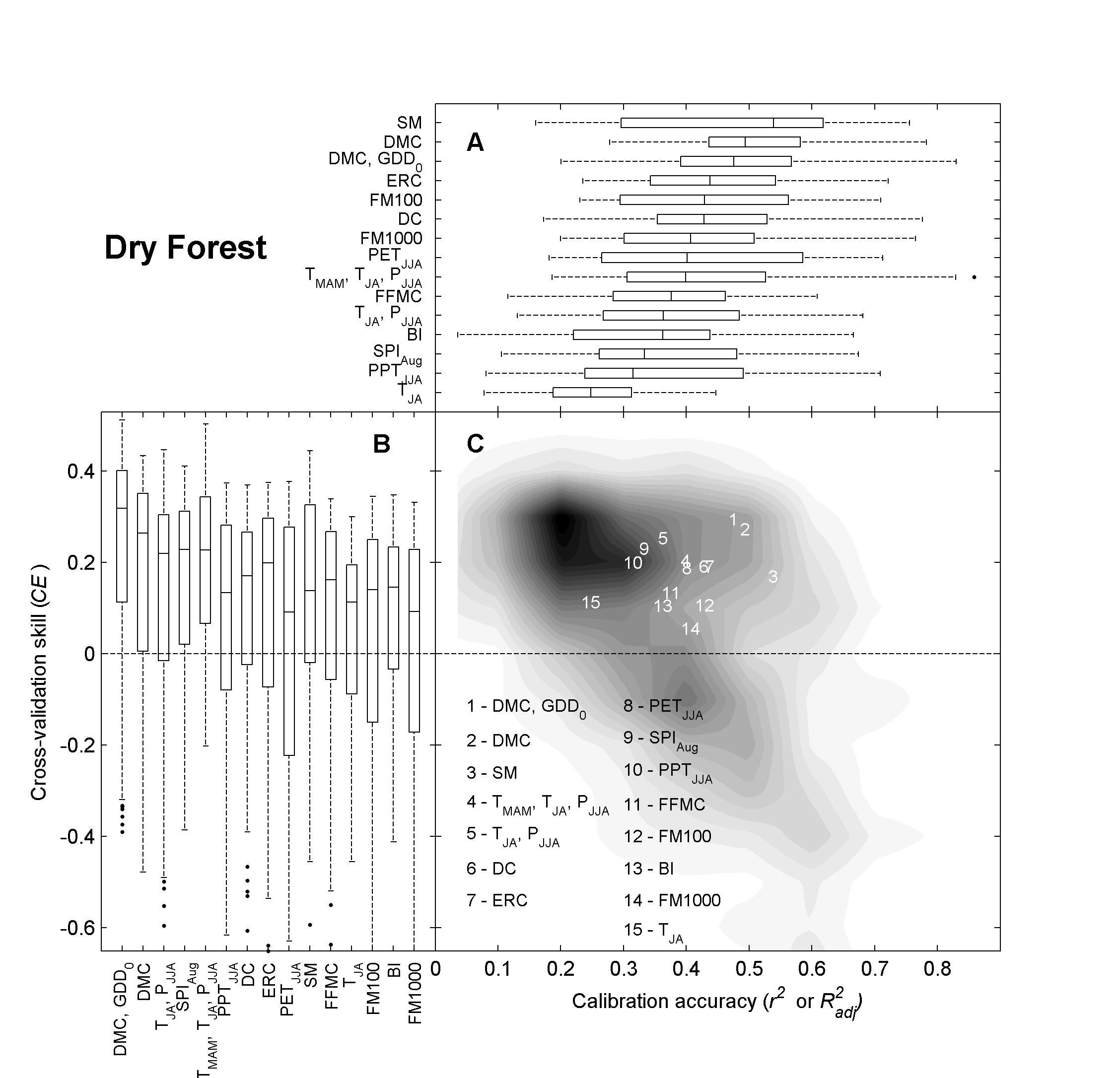


**Figure B. Calibration accuracy and cross-validation skill of potential predictors of annual area burned in Dry Forest sites.** Details are as in Fig. A.


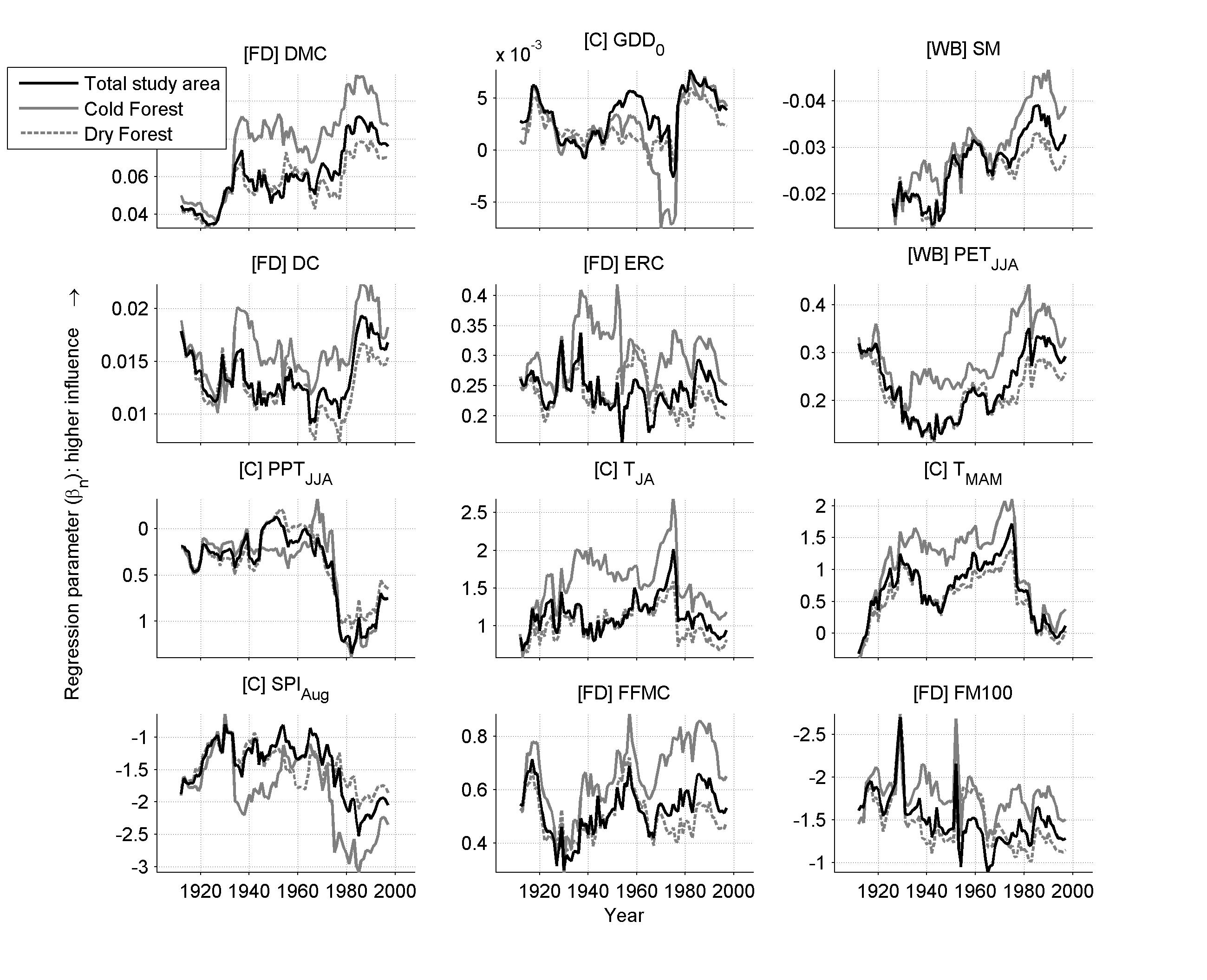


**Figure C. Variability in regression parameters through time, for the total study area and stratified by dominant forest type.** Regression parameters represent the slope of the model, β_1_, for single-variable regression models. GDD_0_ represents β_2_ from the combined DMC, GDD_0_ model, while PPT_JJA_, T_JA_, and T_MAM_ represent β_2_, β_3_, and β_4_ from the three-variable model. Metrics are ordered from upper left to bottom right based on the overall model score (Table 2).

| **Cold Forest**  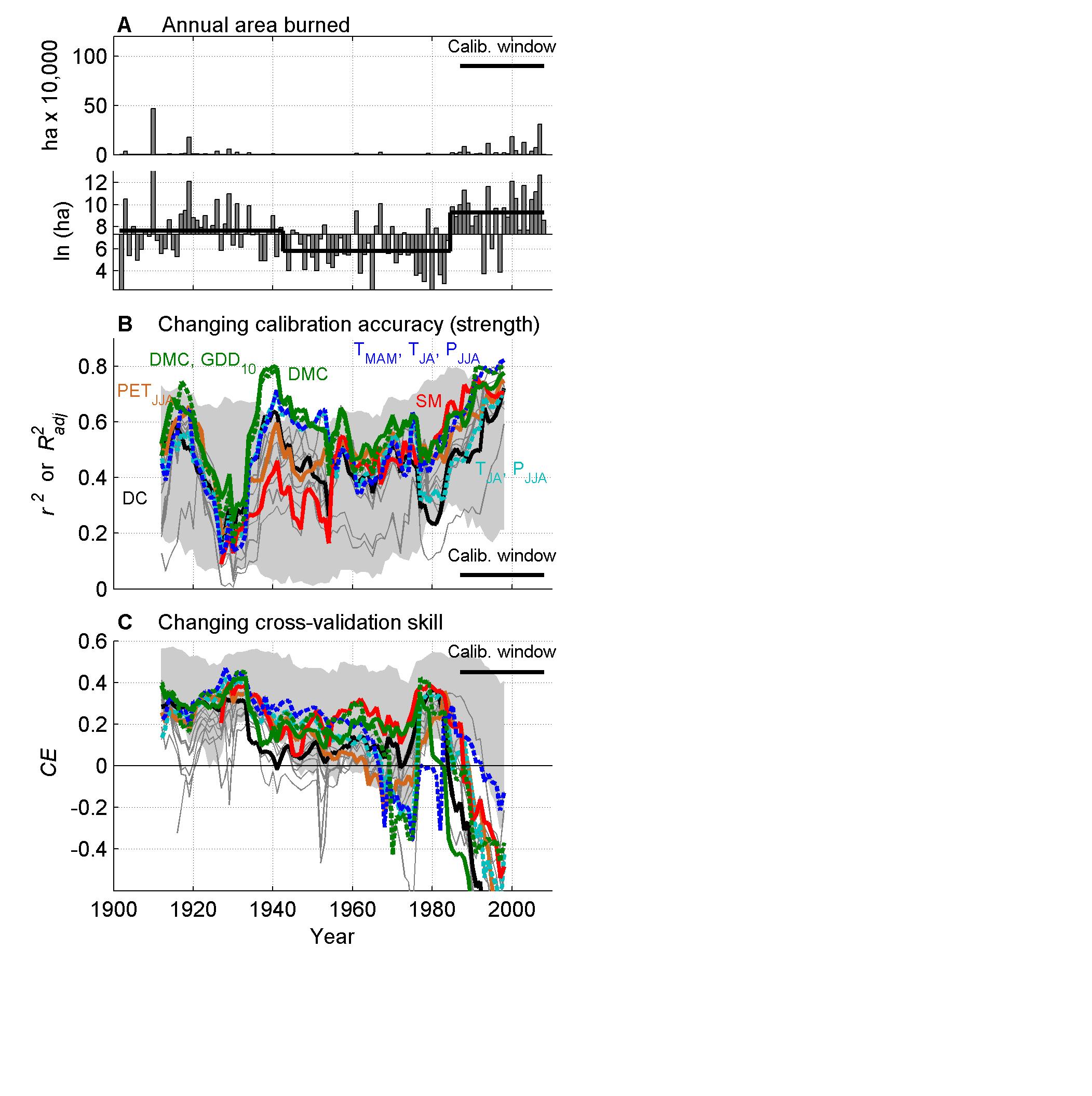 | **Dry Forest**  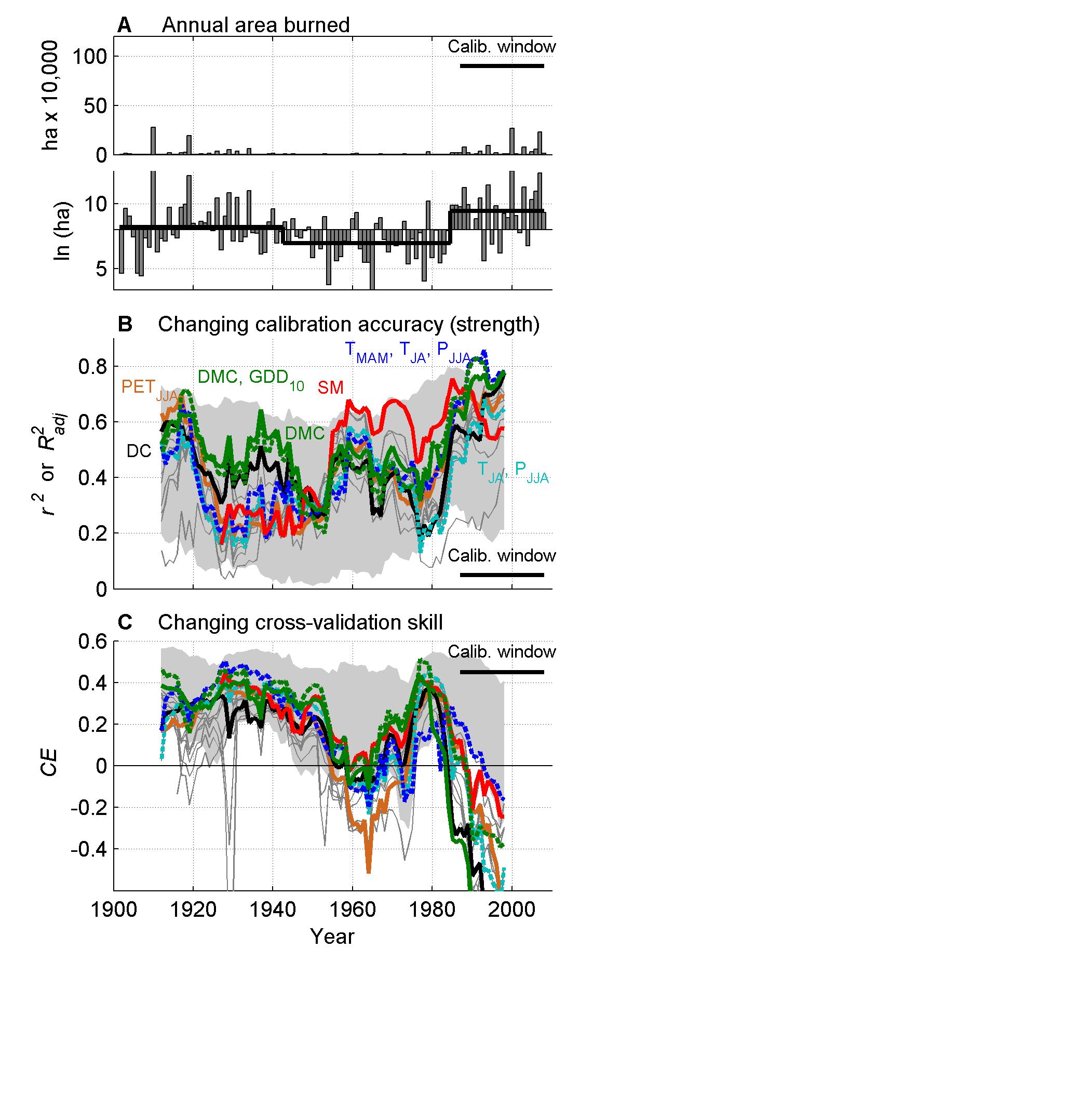 |
| --- | --- |

**Figure D. Variability in the strength and nature of fire-climate relationships through time, for Cold and Dry forests.** (A) Annual area burned in linear and log-transformed space (baseline = series-wide average; thick black line = period averages, as in Fig. 3 in the main text). NOTE: the change point at 1943 was identified in each area burned time series, while the change point in 1985, identified in the study-wide and Dry Forest analyses, was identified in 1983 in the Cold Forest analysis. For simplicity, the same change points are used in both records. (B) The changing strength of fire-climate relationships (*r^2^* or *R^2^_adj_*) for each 21-yr model. Metrics with the highest explanatory power are labeled, and the length of each overlapping calibration period is represented in the lower right of the panel (“Calib. window”). (C) Changing skill of fire-climate relationships, *CE*, as in (B).

**Middle Rockies ecoprovince**


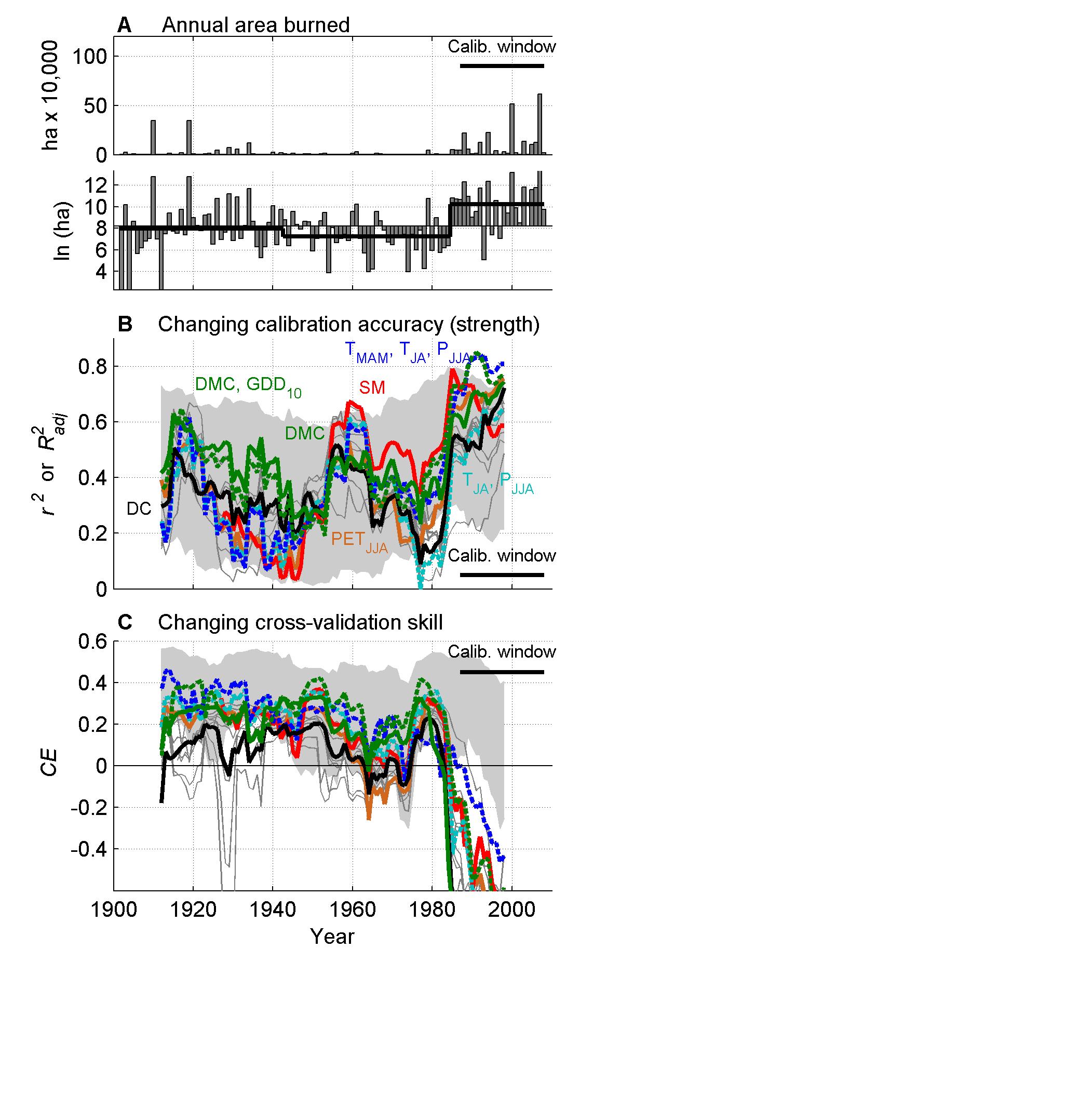


**Figure E. Variability in the strength and nature of fire-climate relationships through time, for the Middle Rockies ecoprovince.** See Fig. D for explanation.


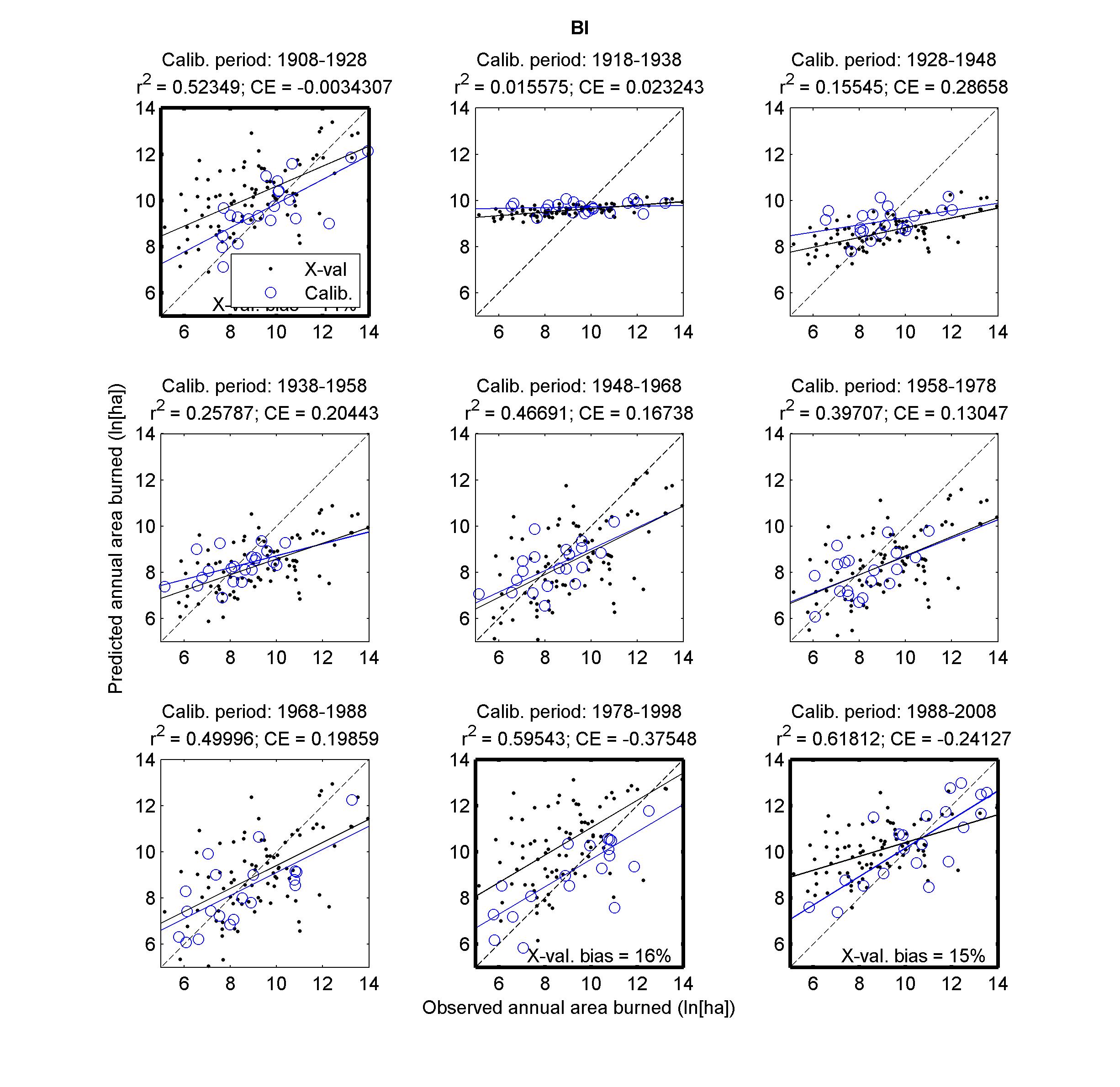

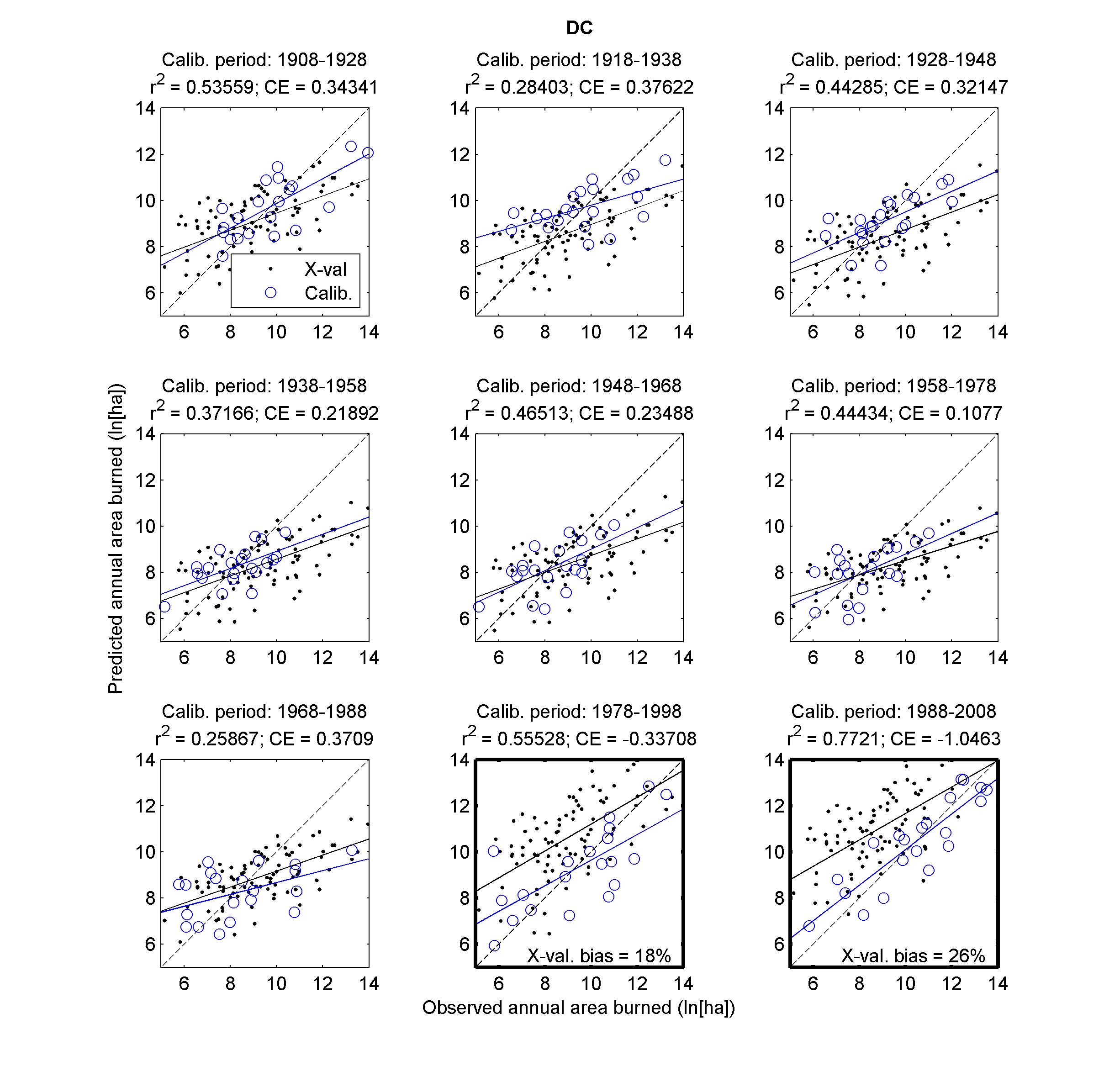

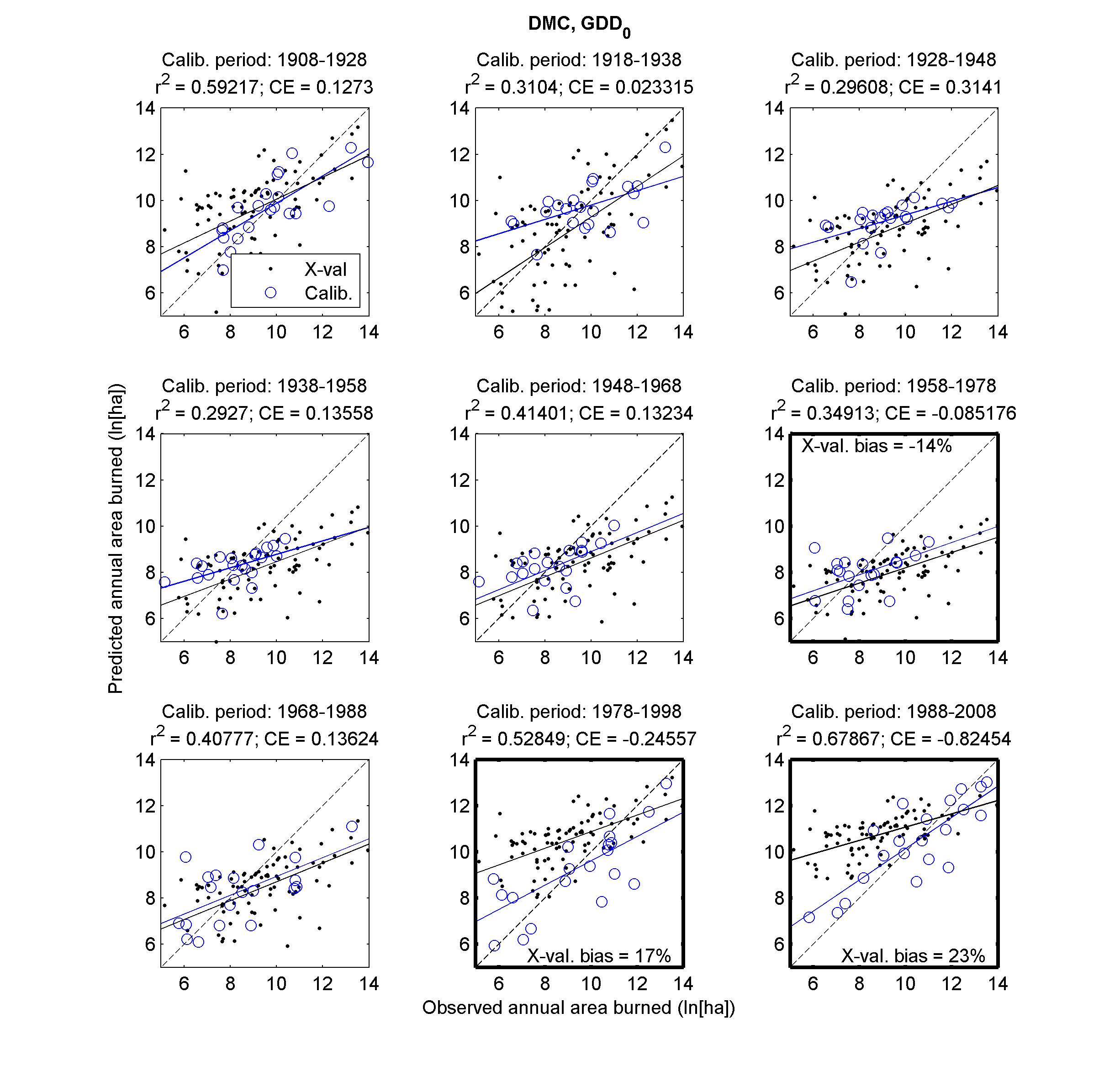

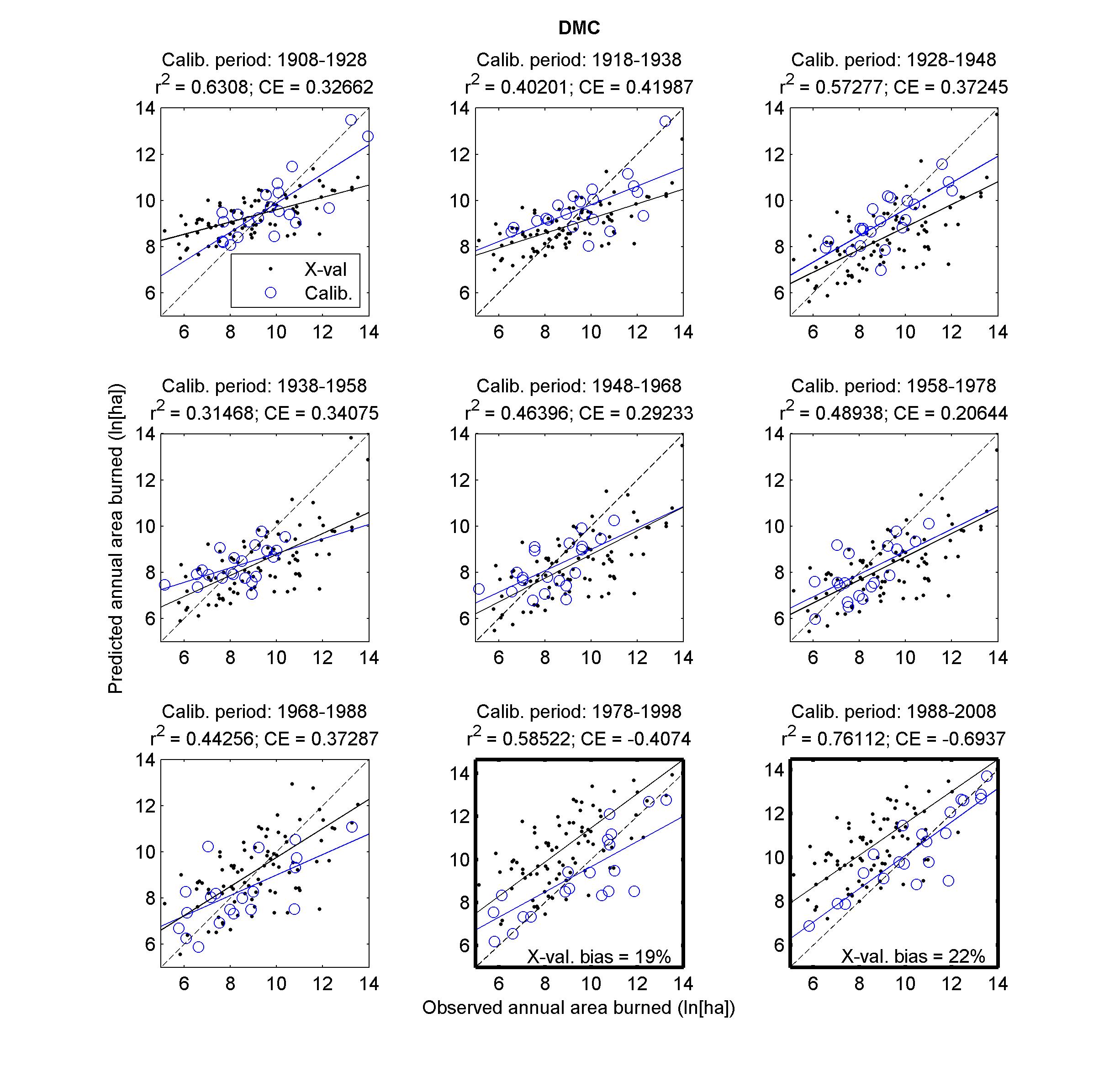

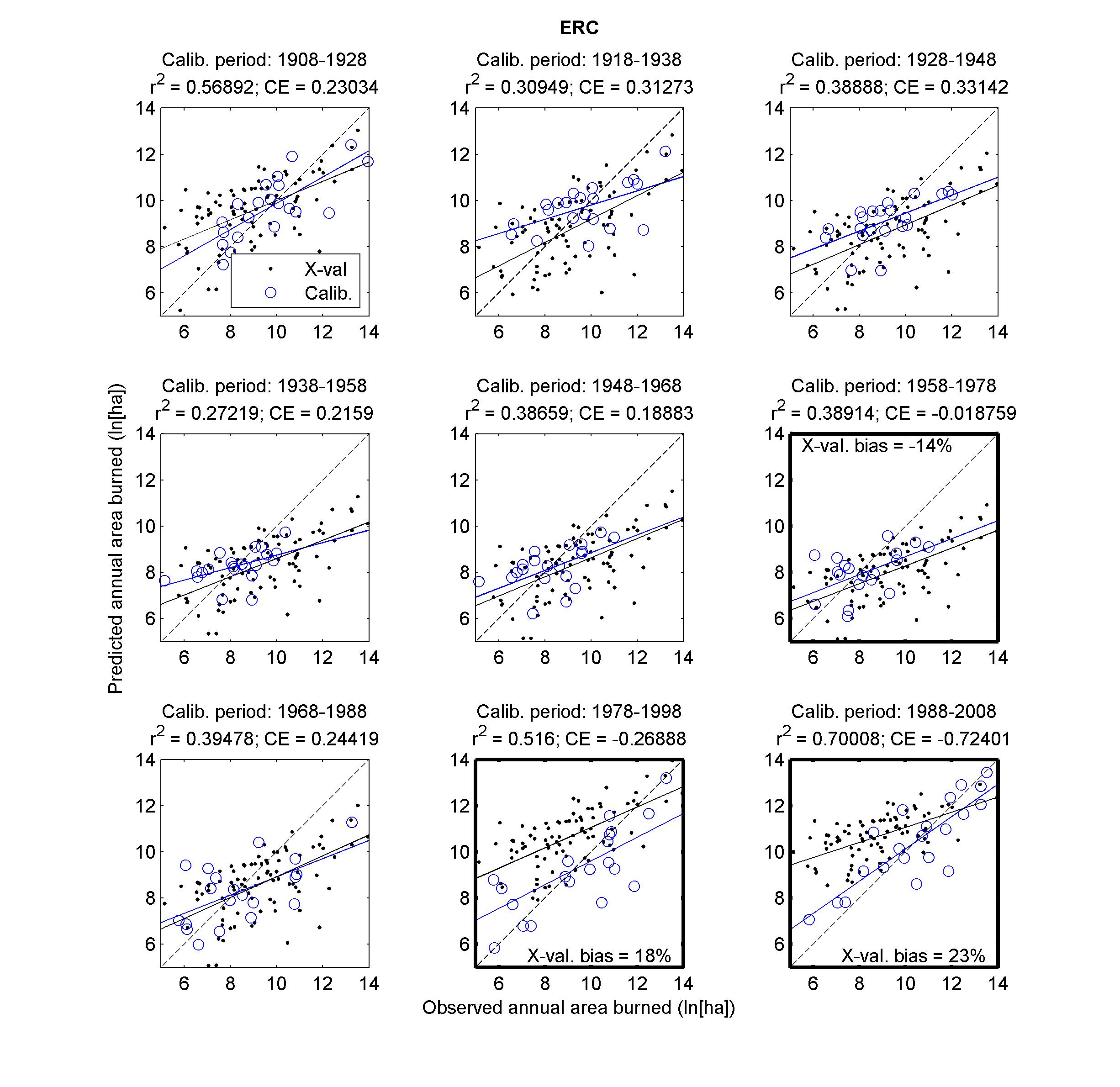

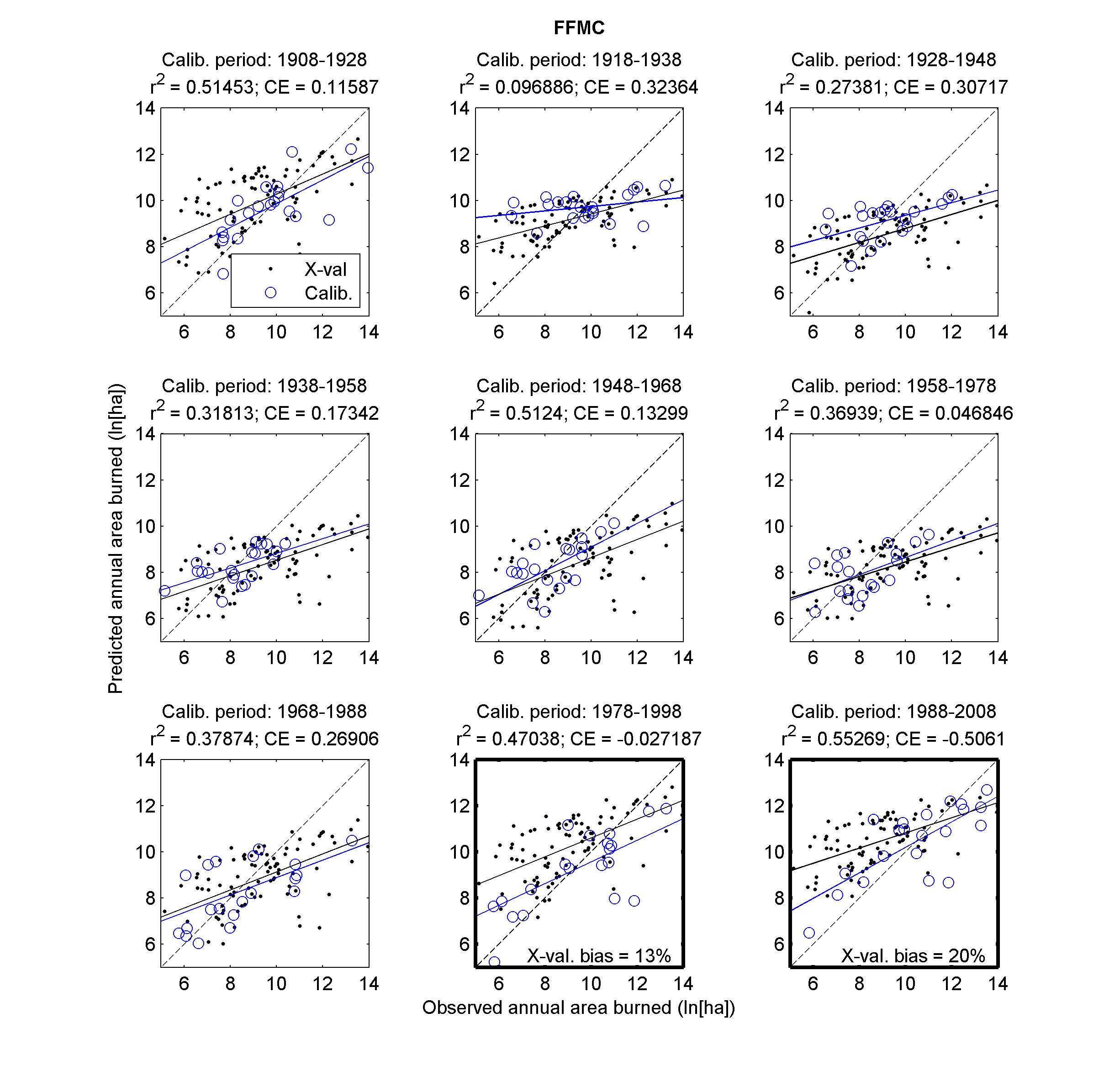

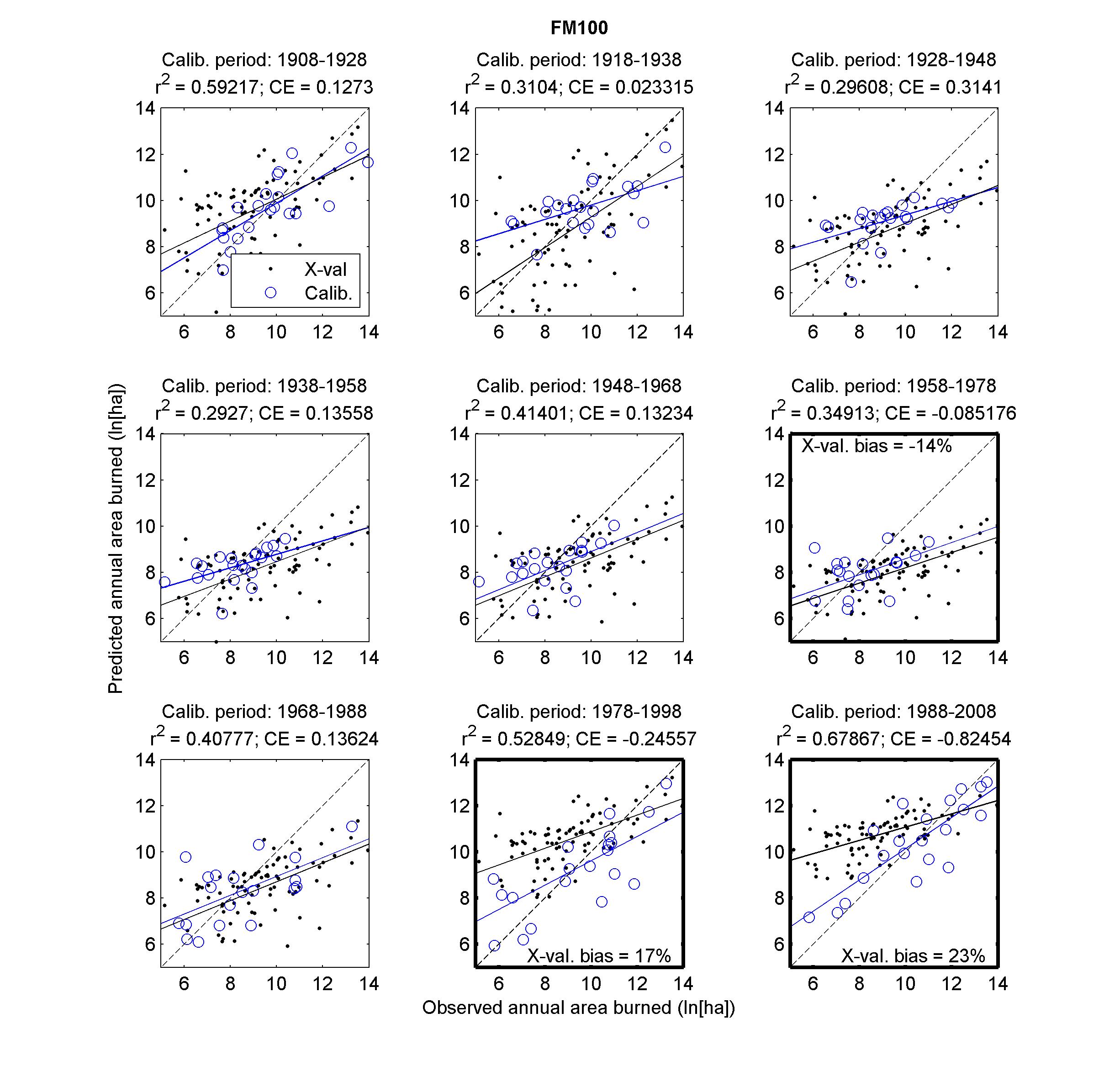

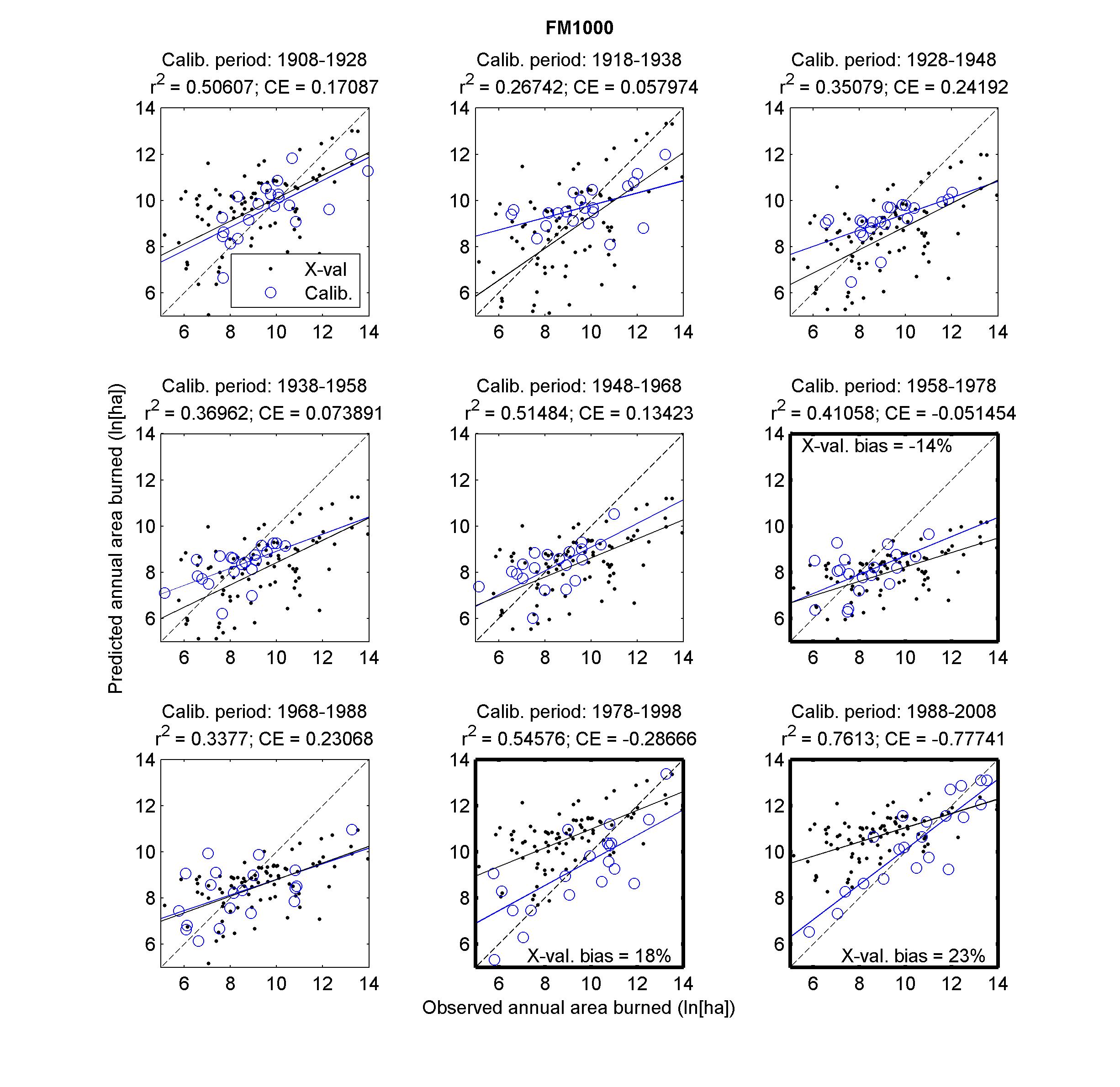

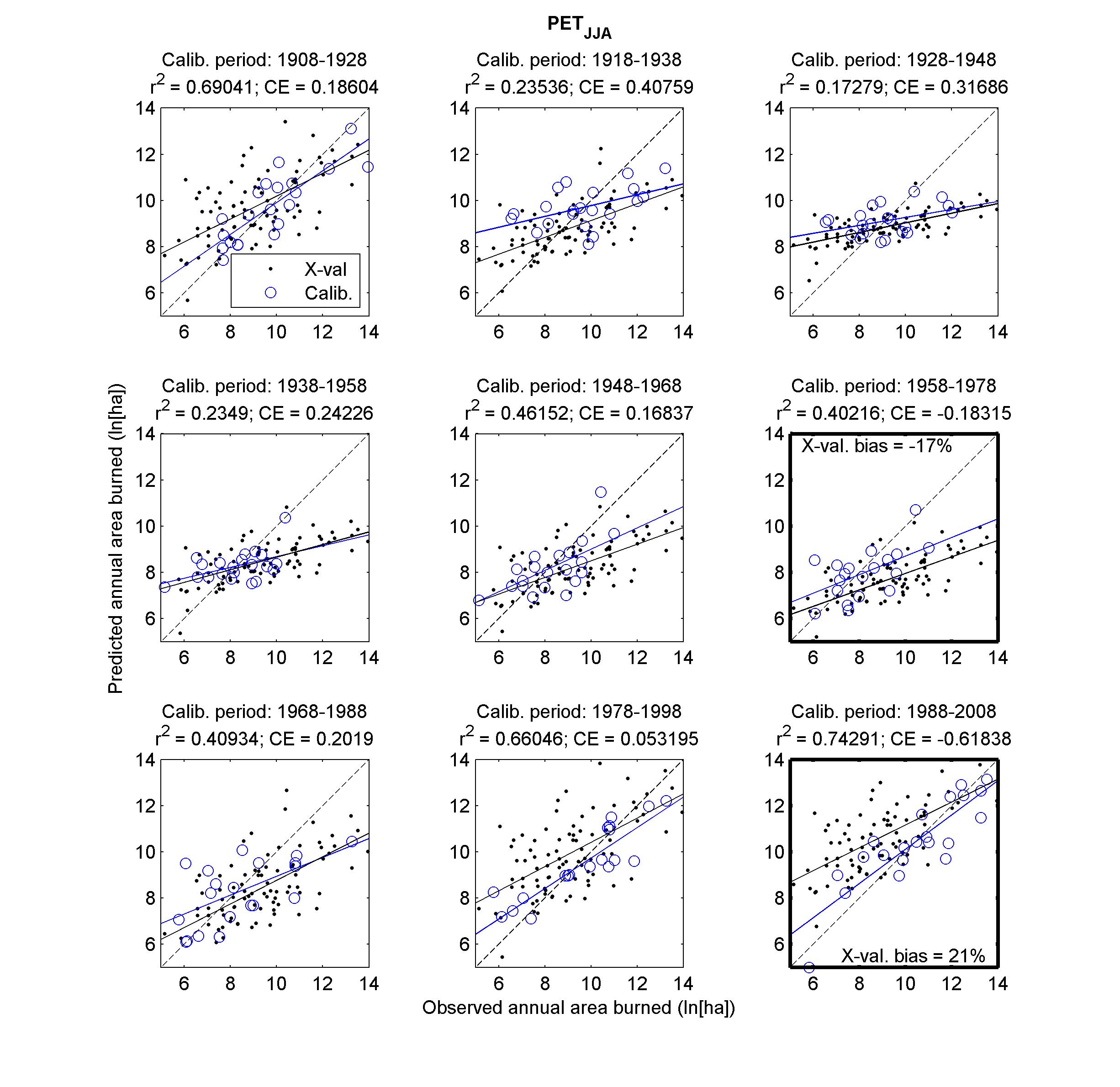

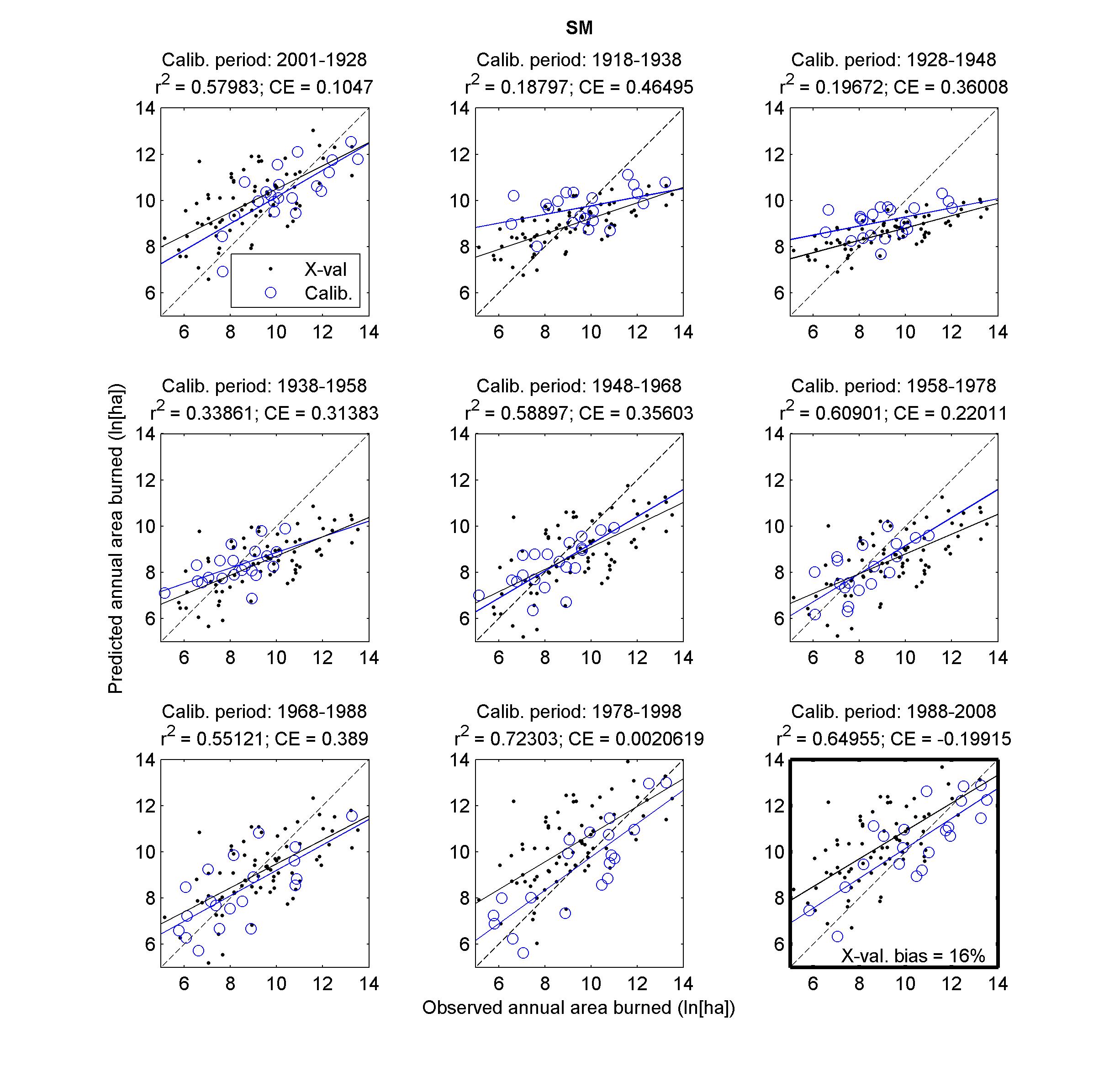

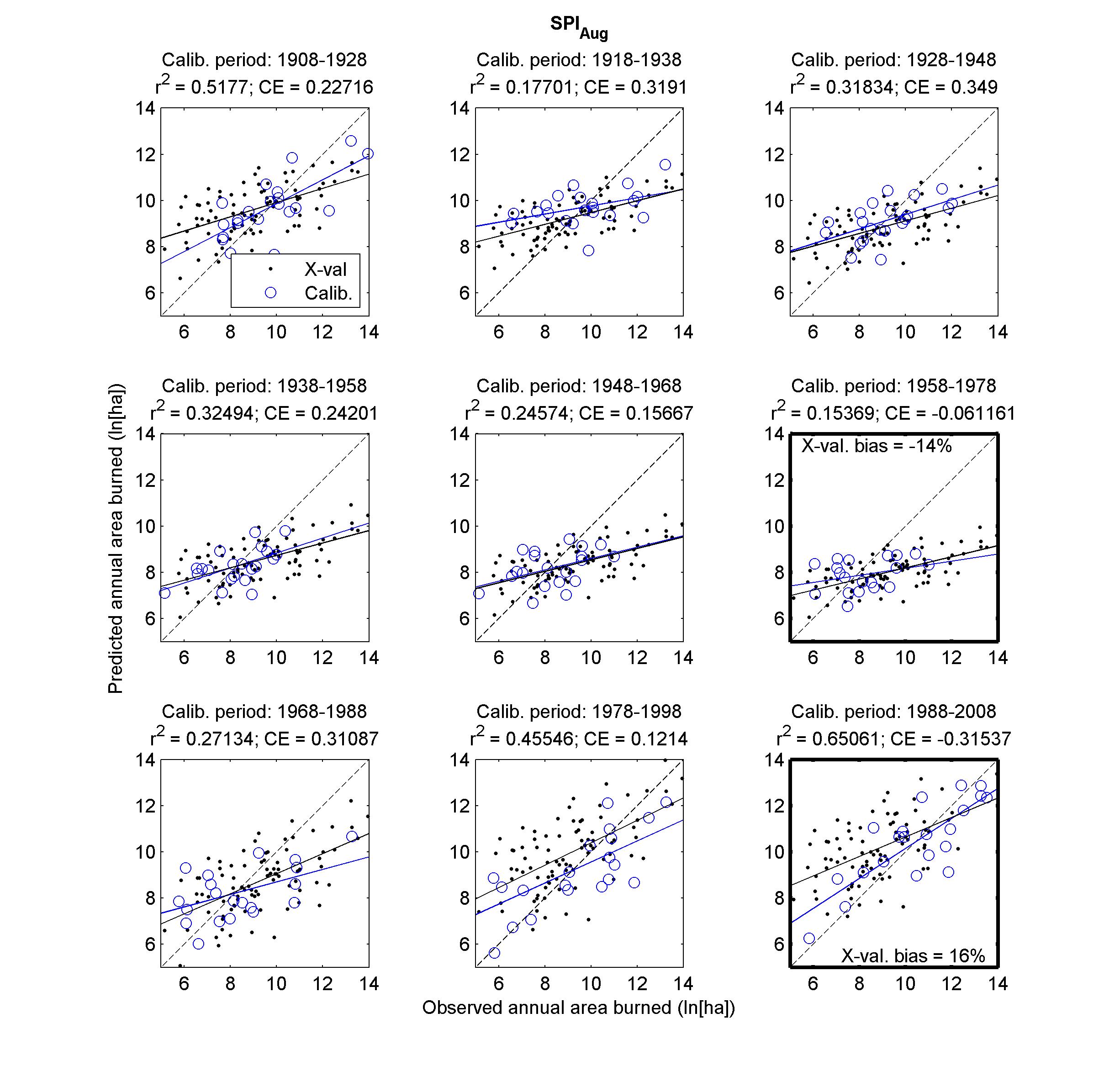

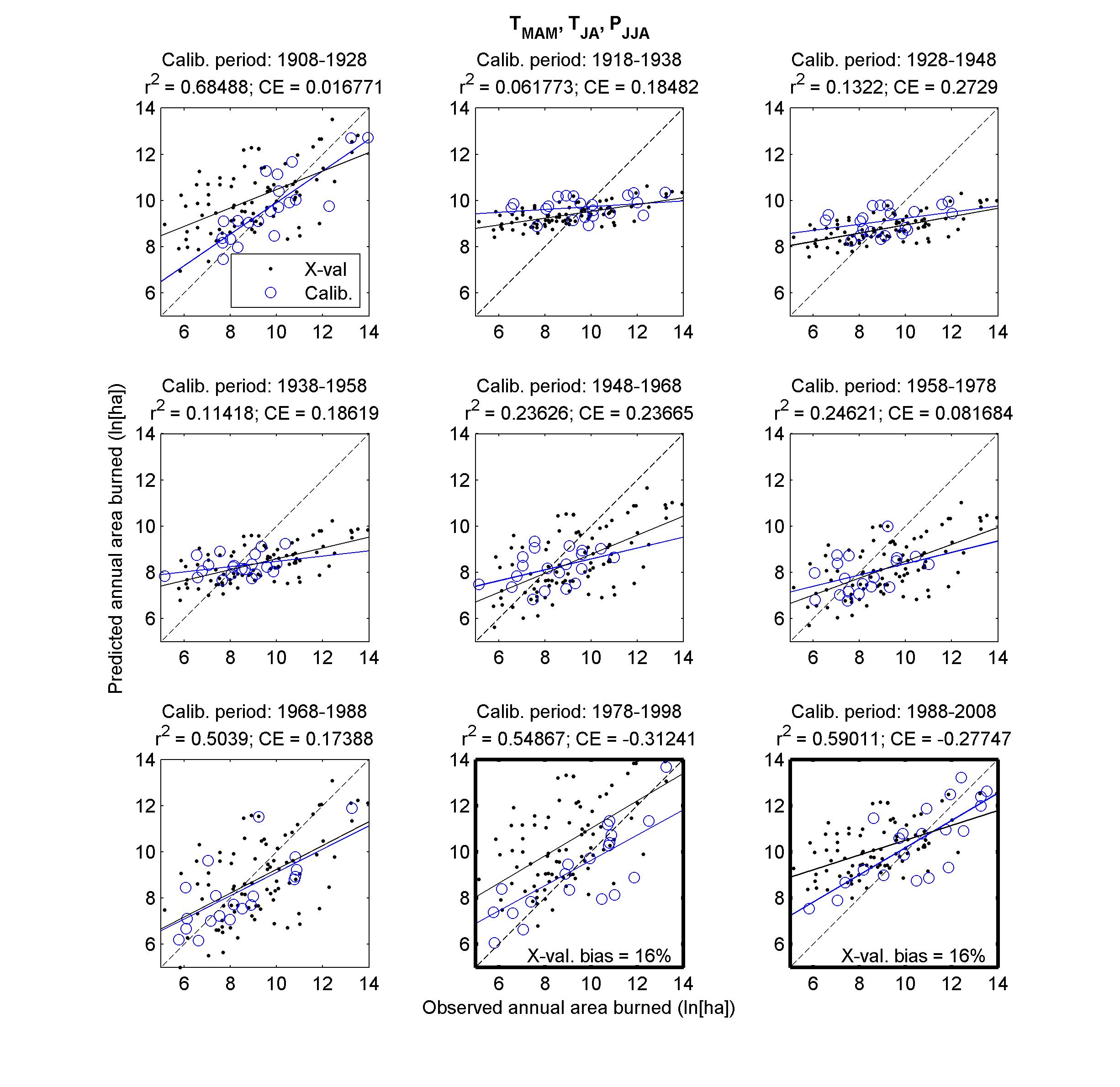


**Figure F. Time-varying calibration accuracy and cross validation skill of fire-climate models.** Predicted annual area burned (y-axis) is plotted as a function of observed annual area burned (x-axis), for both the calibration the period (“Calib.”) and cross-validation period (“X-val”). Each panel represents a subset of all analyses, staggered by ten years, and includes the dates of the calibration period, the calibration period accuracy (*r^2^*), and the cross-validation period skill (*CE*). Periods with no cross-validation skill (i.e., *CE* < 0) are identified by bold subplots, and the average bias in cross-validation predictions is noted within the subplot. The specific climate metric plotted is noted at the top of each page. Models are in alphabetical order.
